# Supplementary figures and images for: FAM83F regulates canonical Wnt signalling through an interaction with CK1α
Source: Life Sci Alliance. 2020 Dec 24;4(2):e202000805. doi: 10.26508/lsa.202000805 (PMC7768192; doi:10.26508/lsa.202000805)

Figure 1D.

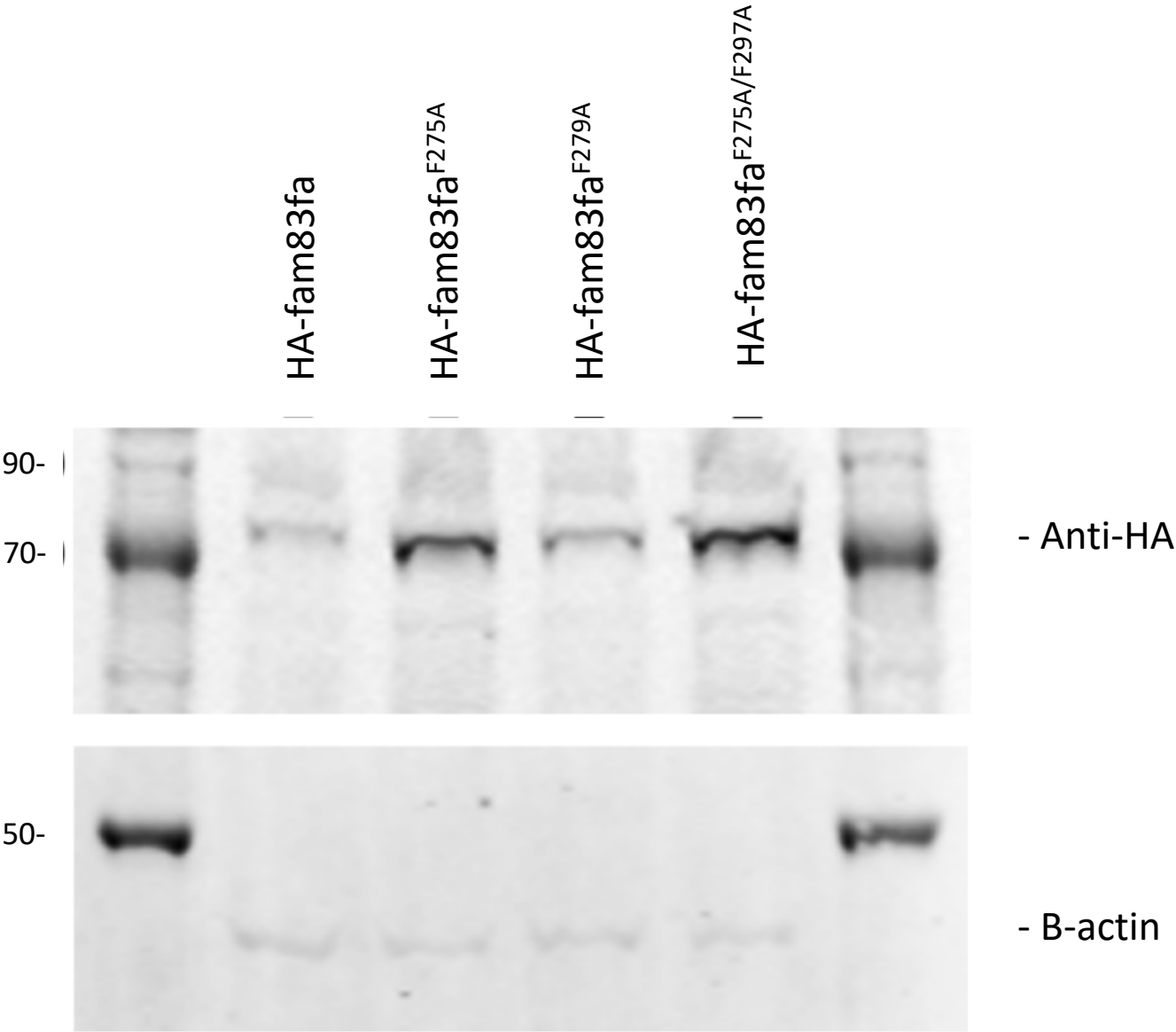

Supplement: Supplementary file 1 [file LSA-2020-00805_SdataF1.pdf]

Supplementary Figure 2B.

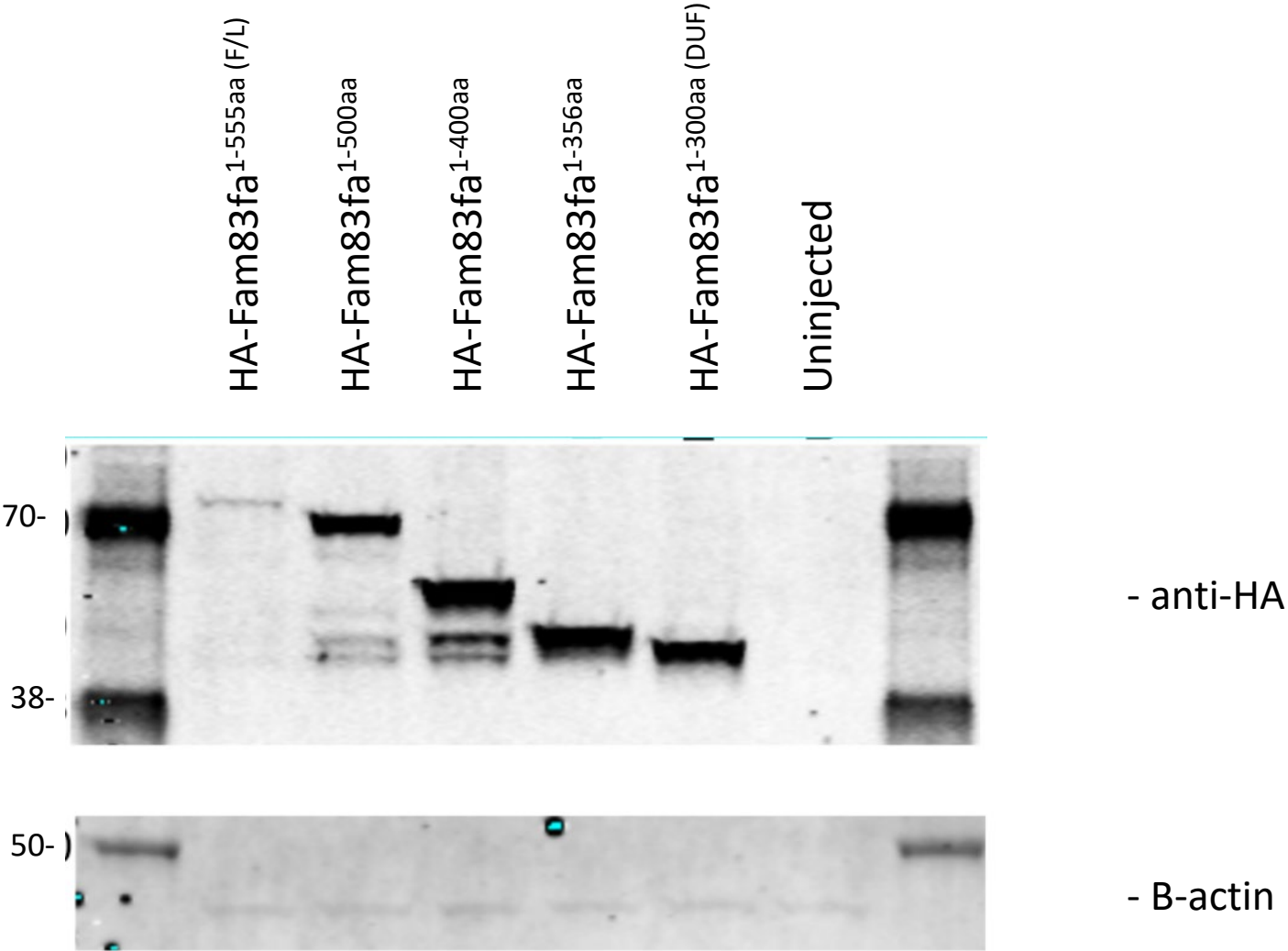

Supplement: Supplementary file 2 [file LSA-2020-00805_SdataFS2.pdf]

Figure 2B.

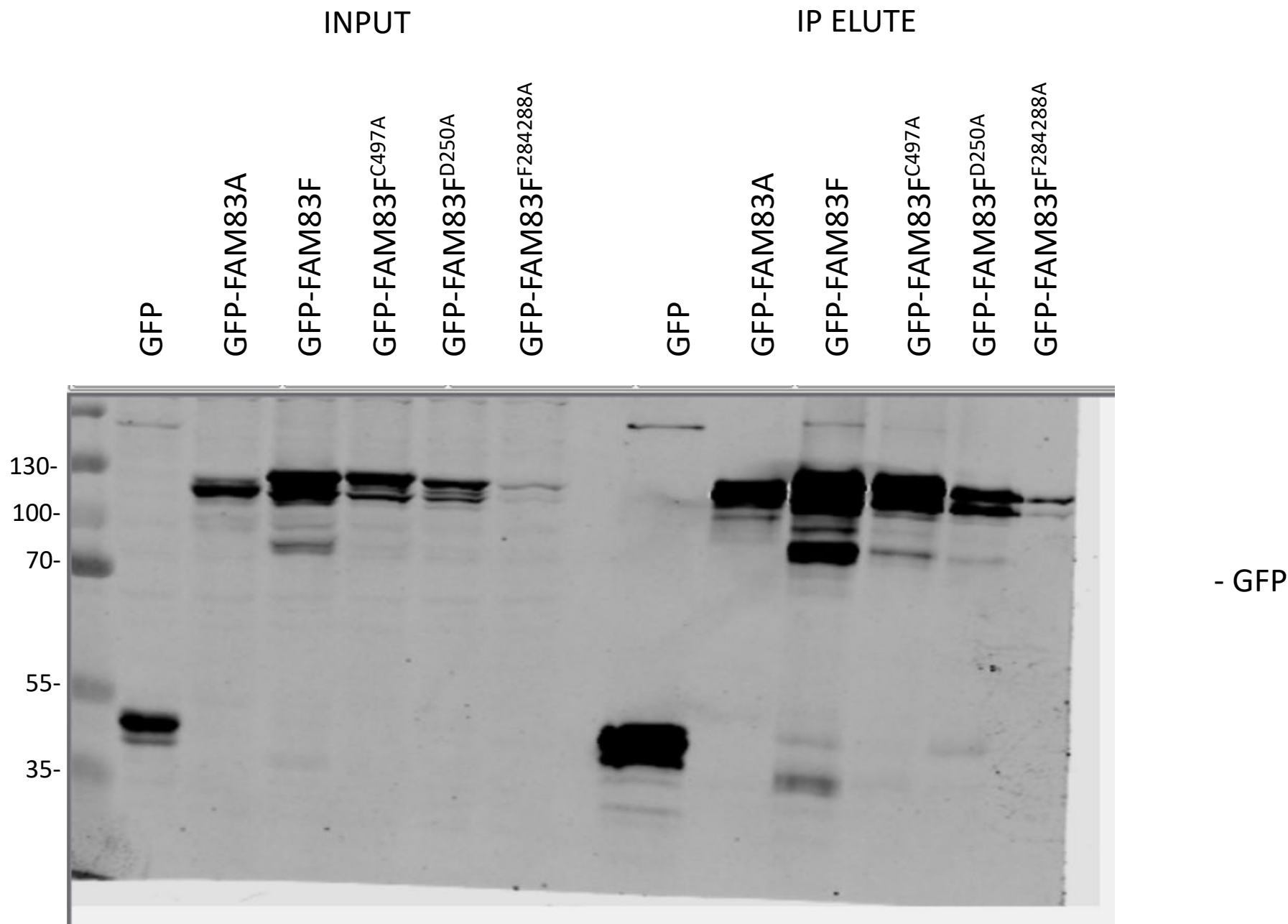

Figure 2B.

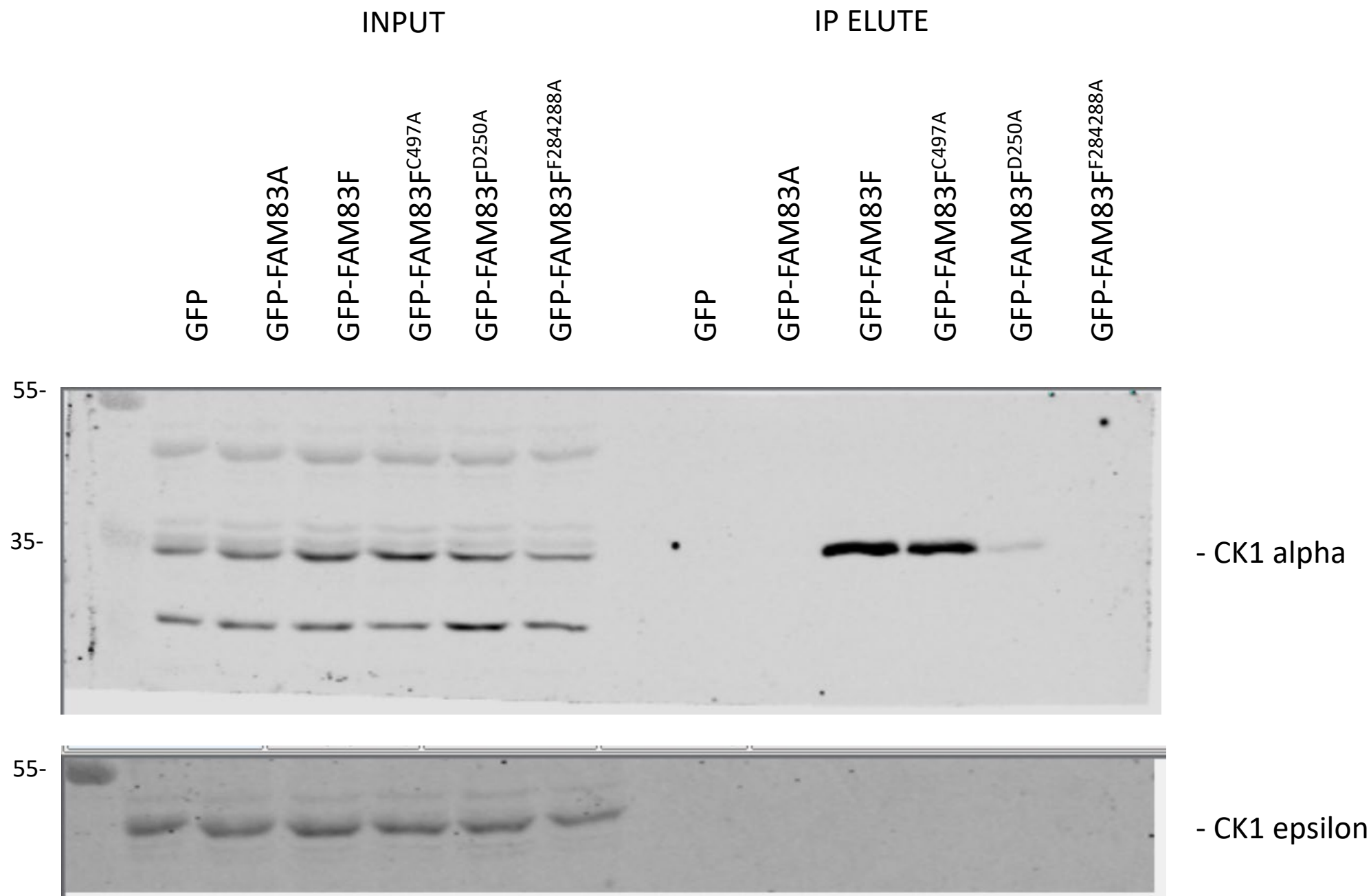

Figure 2B.

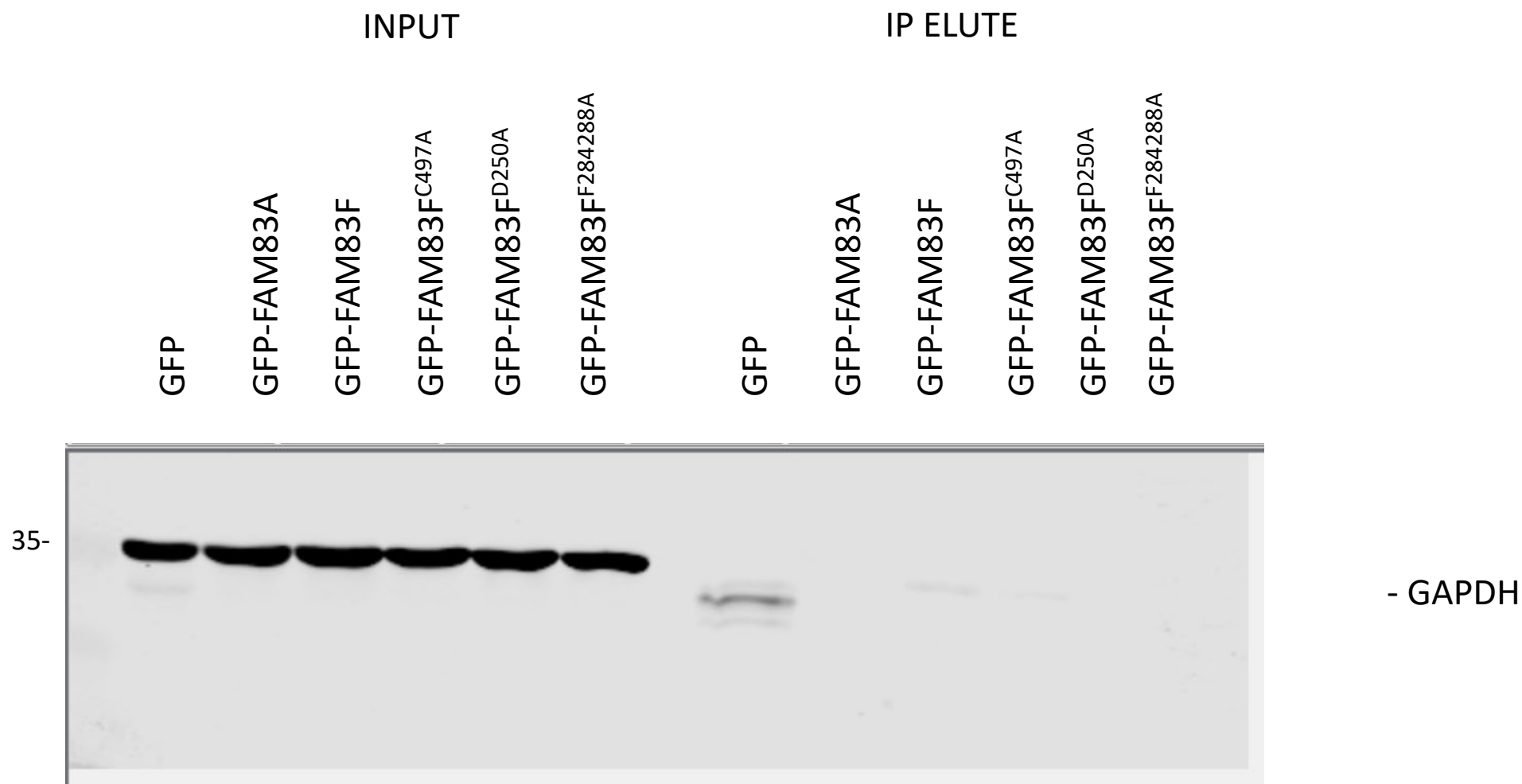

Supplement: Supplementary file 3 [file LSA-2020-00805_SdataF2.pdf]

Figure 3A.

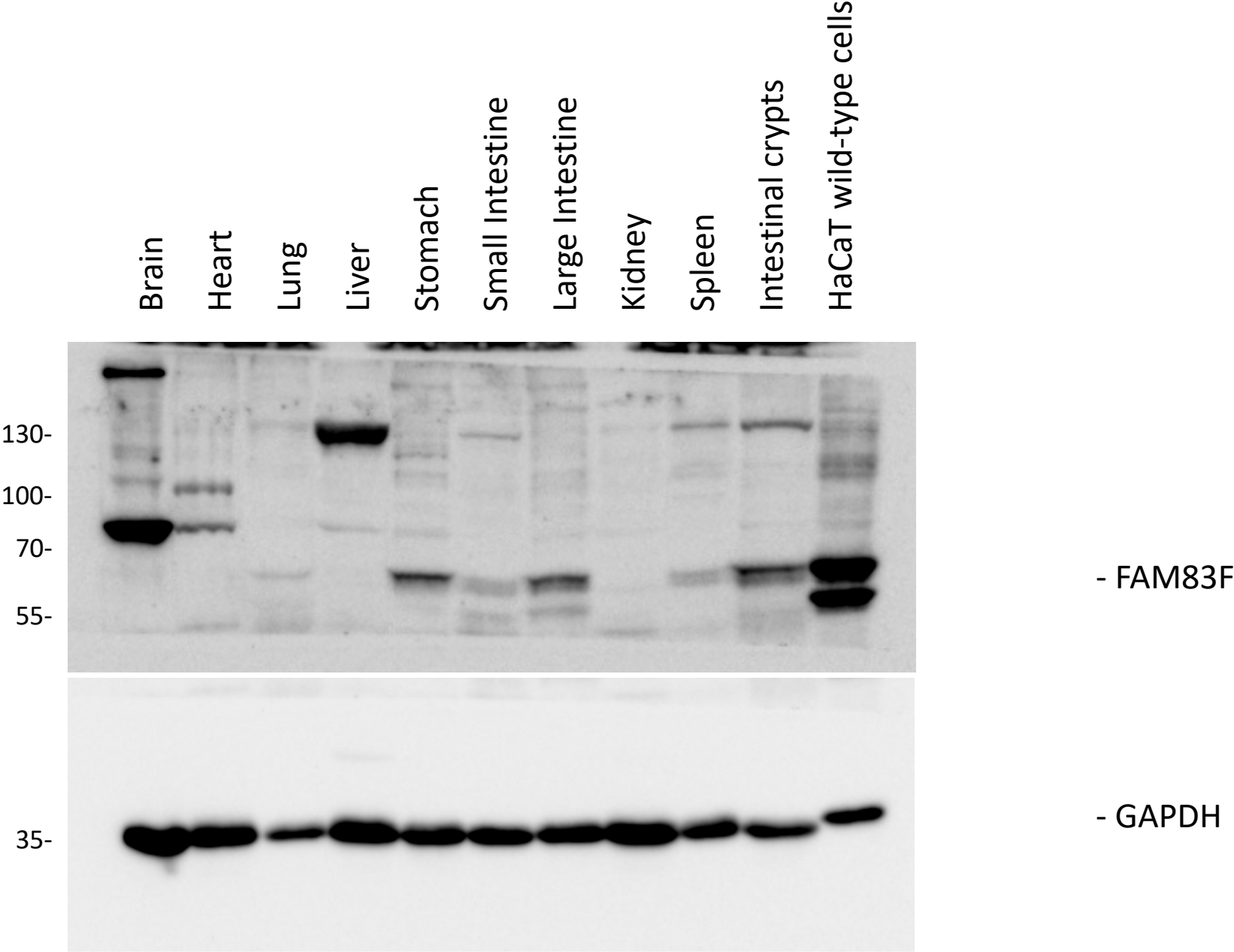

Figure 3B.

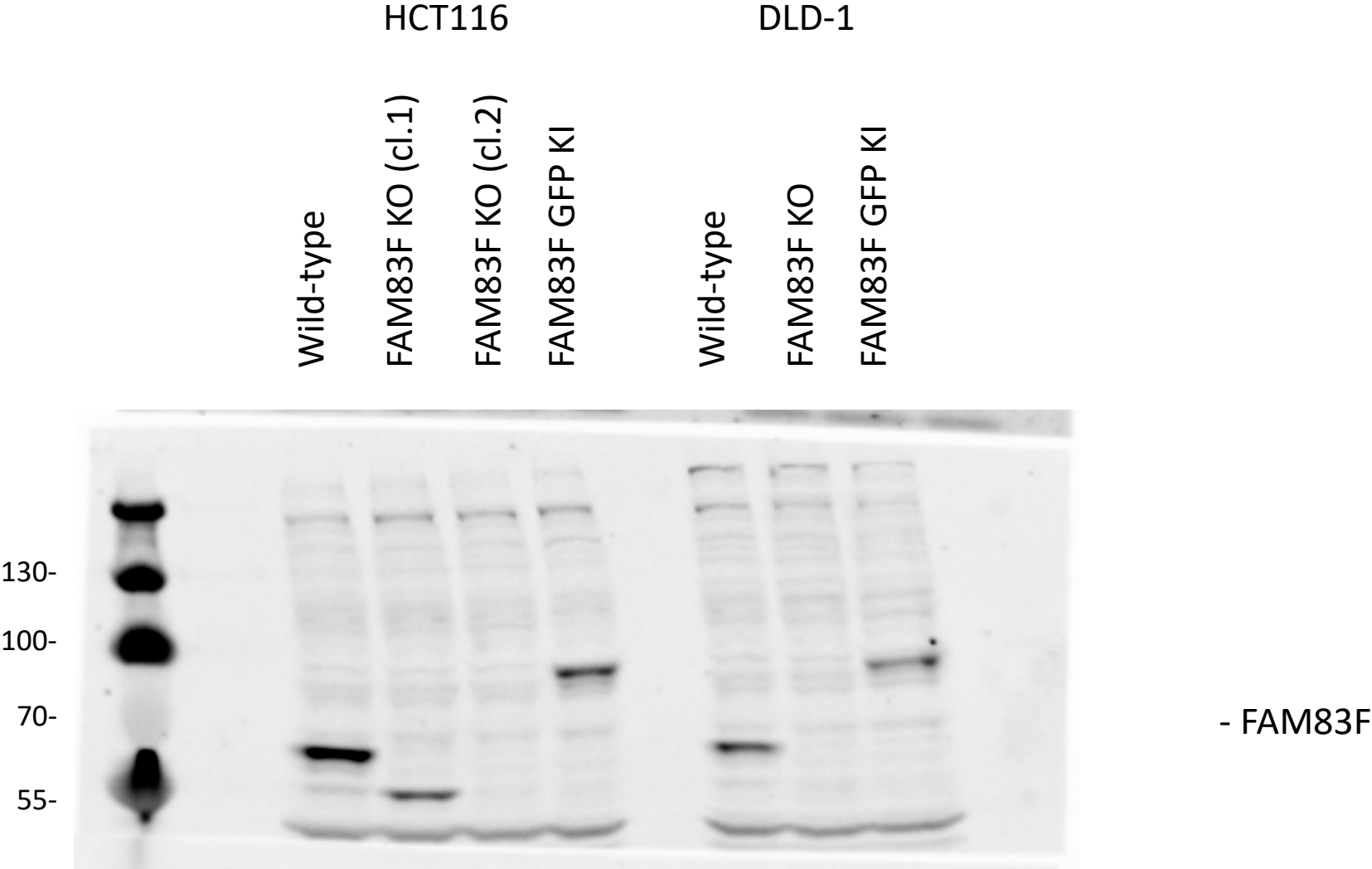

Figure 3B.

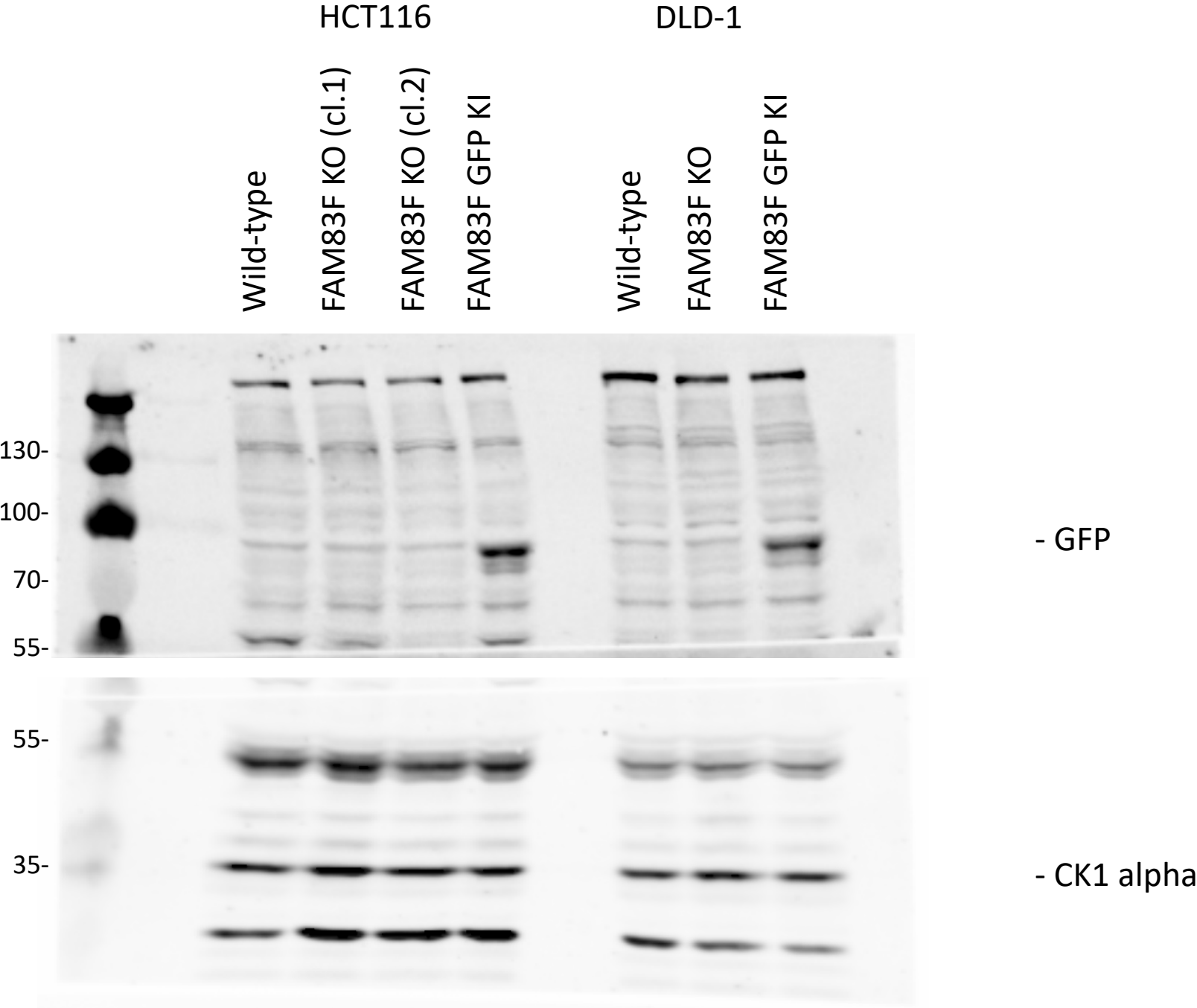

Figure 3B.

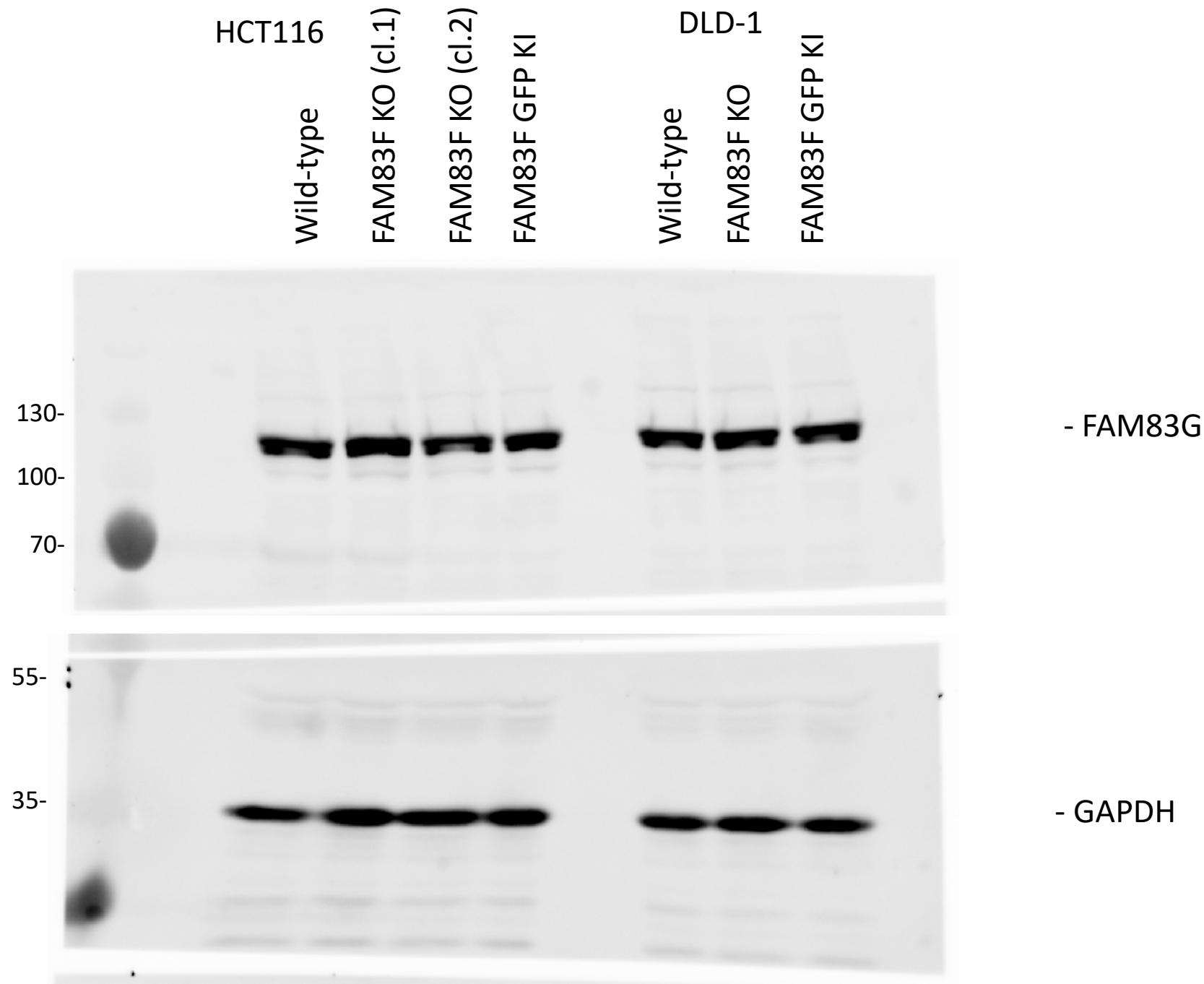

Figure 3C.

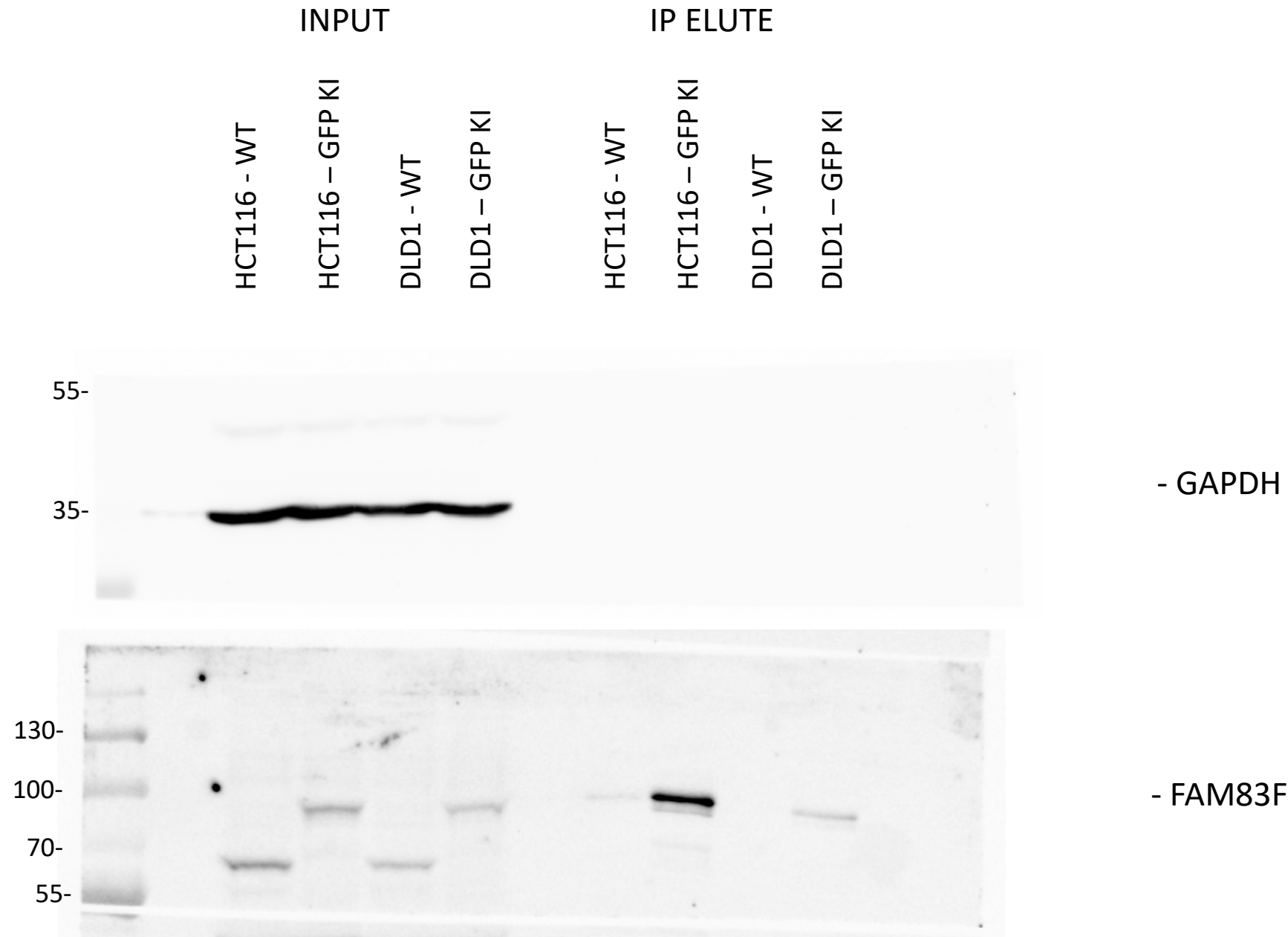

Figure 3C.

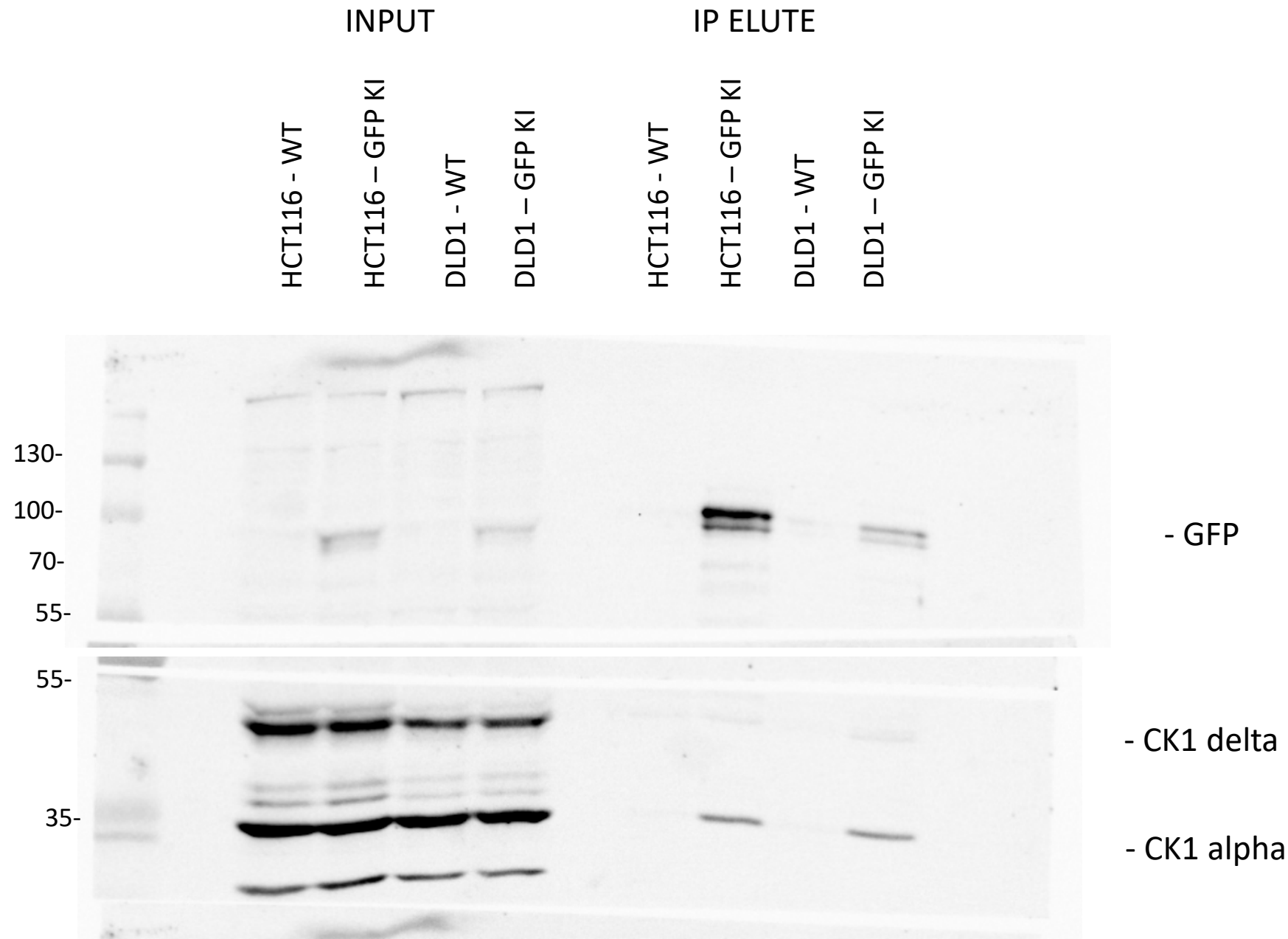

Figure 3C.

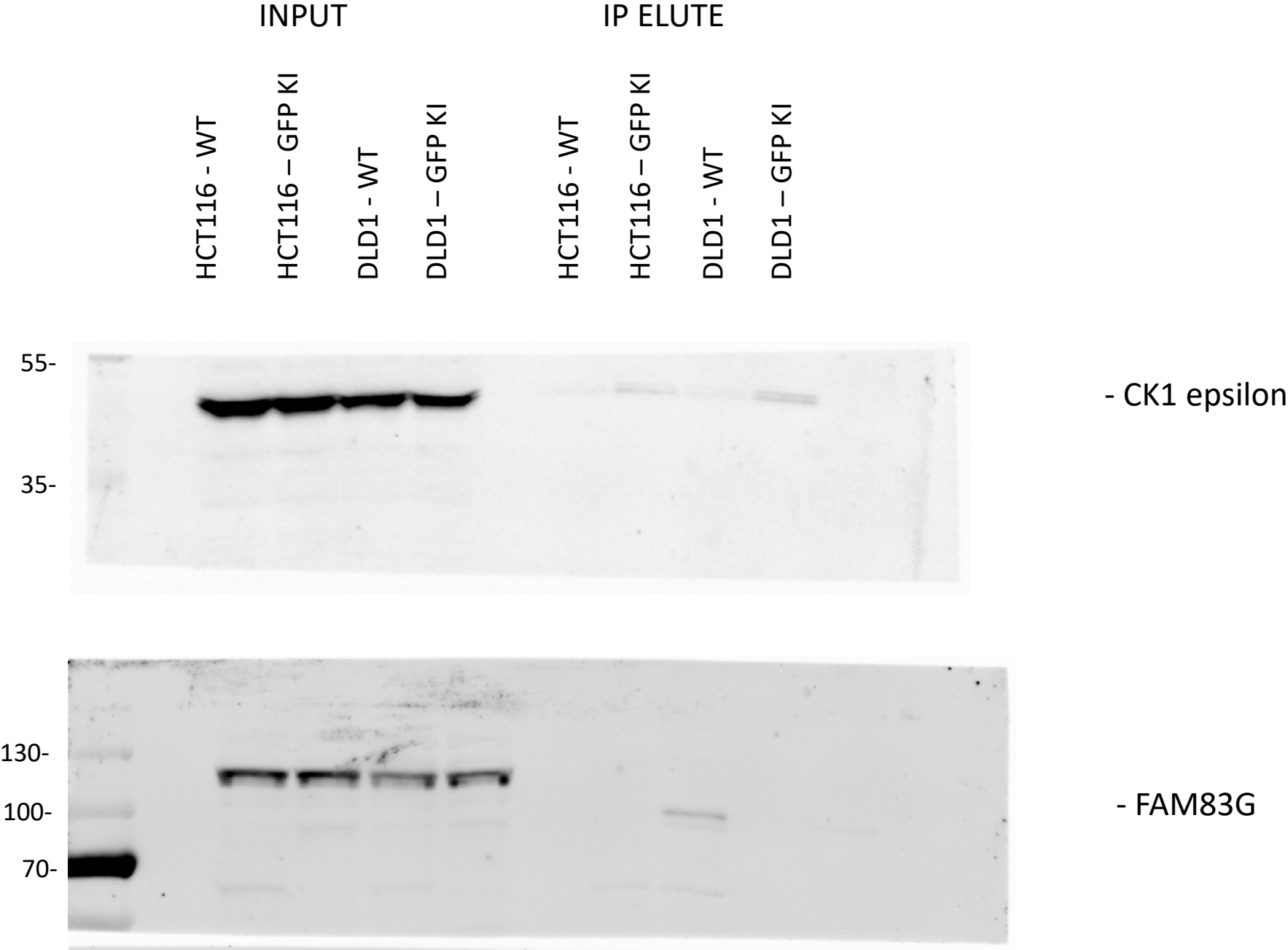

Figure 3D.

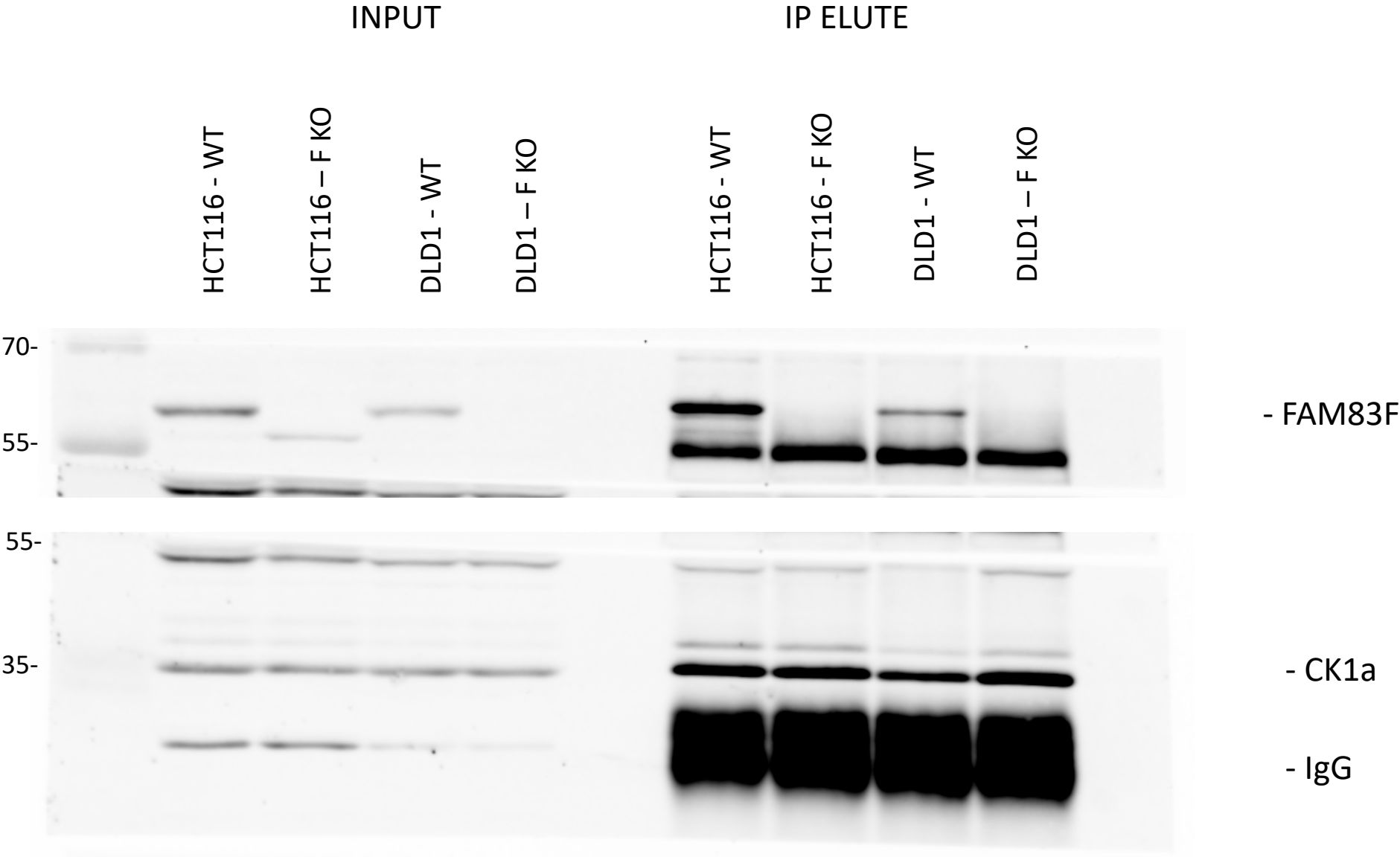

Figure 3D.

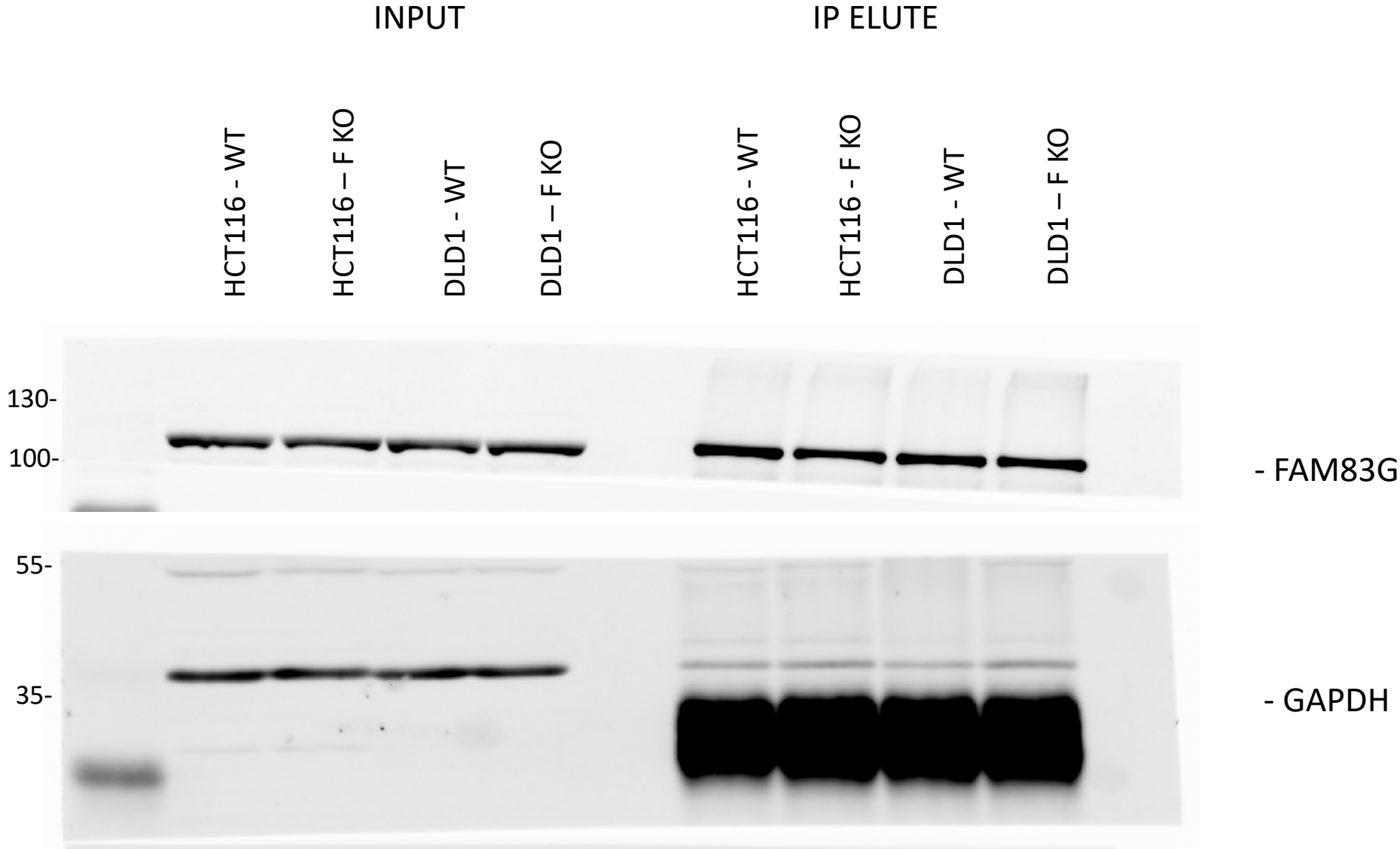

Supplement: Supplementary file 6 [file LSA-2020-00805_SdataF3.pdf]

Supplementary Figure 6A.

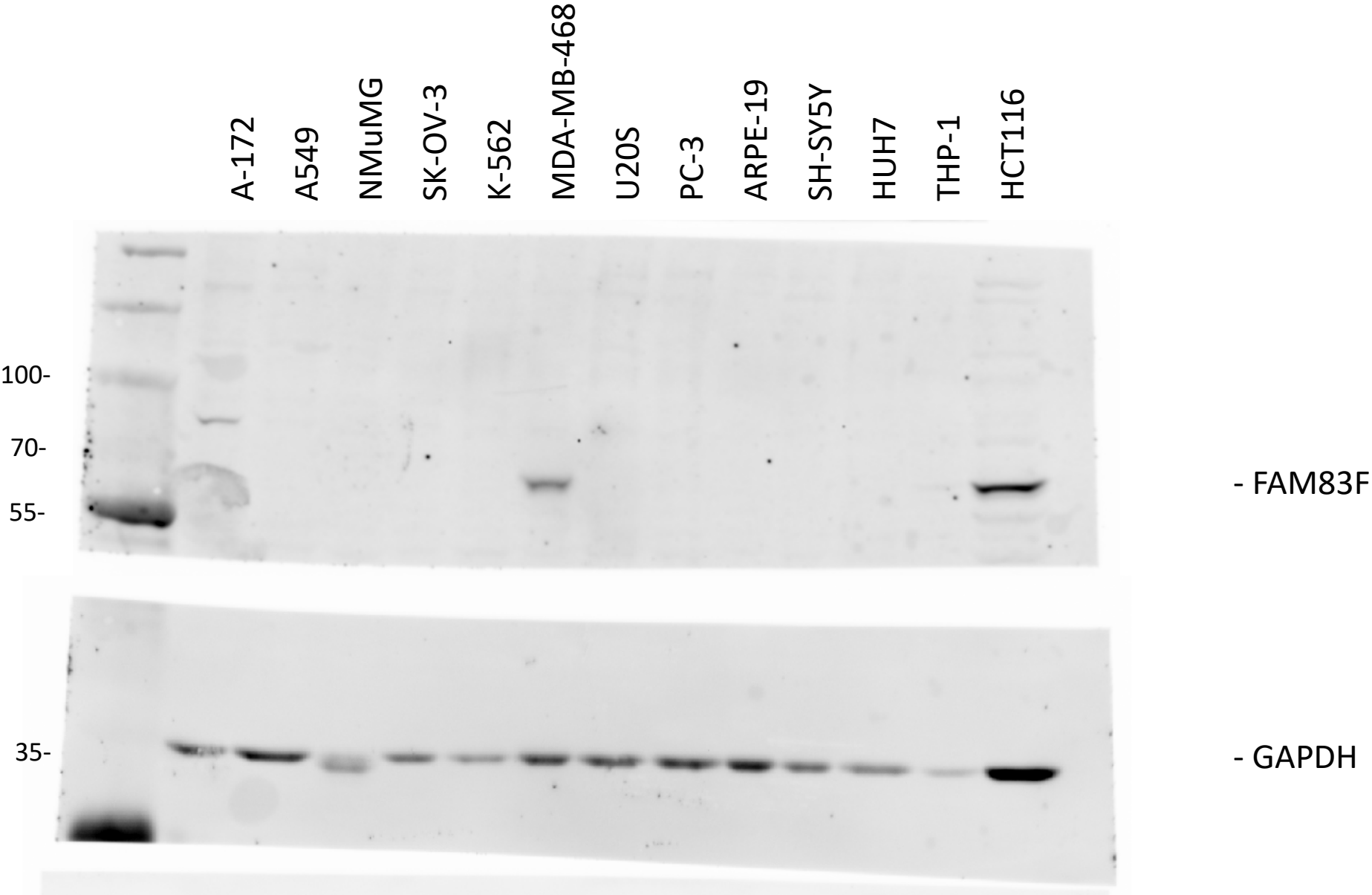

Supplement: Supplementary file 7 [file LSA-2020-00805_SdataFS6.pdf]

Supplementary Figure 9A.

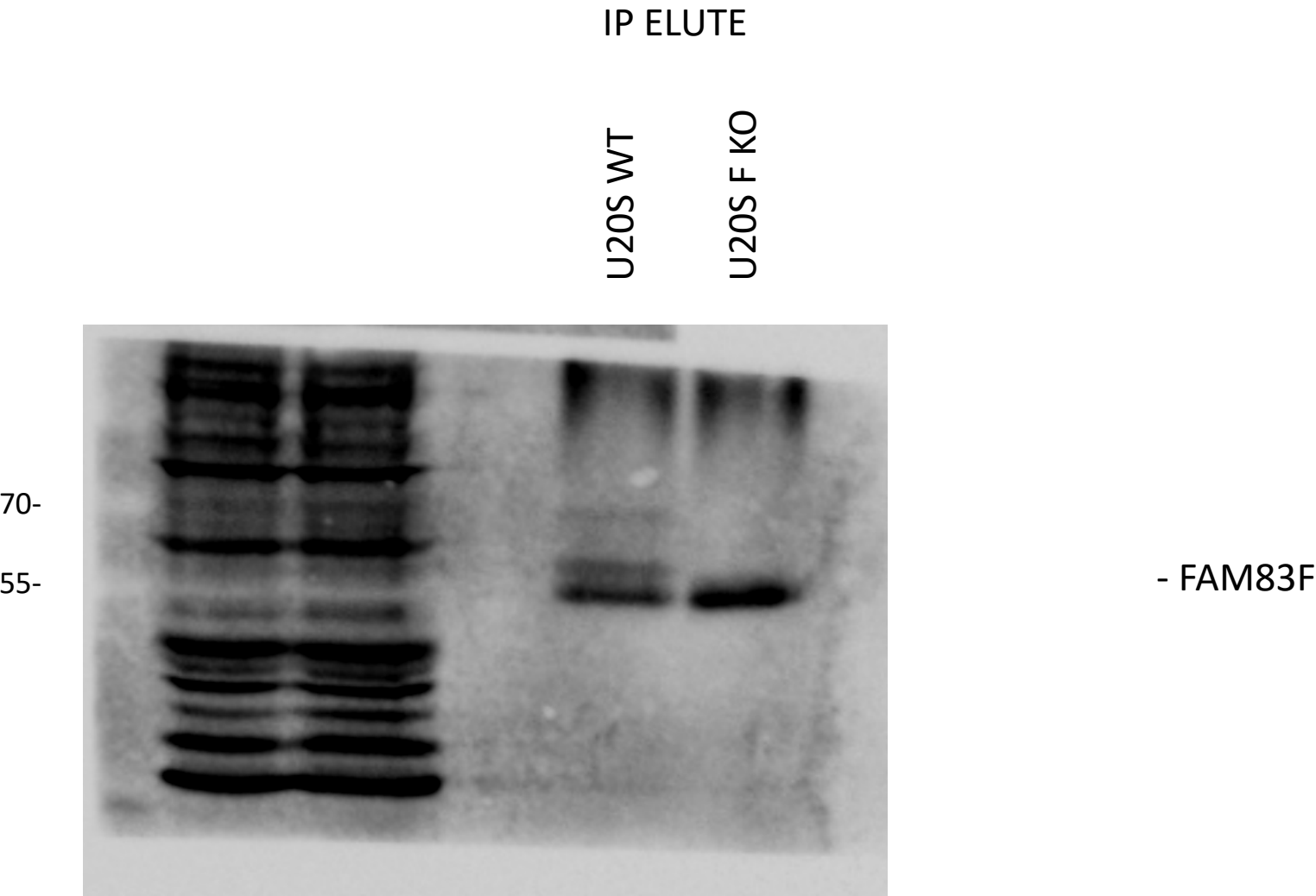

Supplement: Supplementary file 9 [file LSA-2020-00805_SdataFS9.pdf]

Figure 5B.

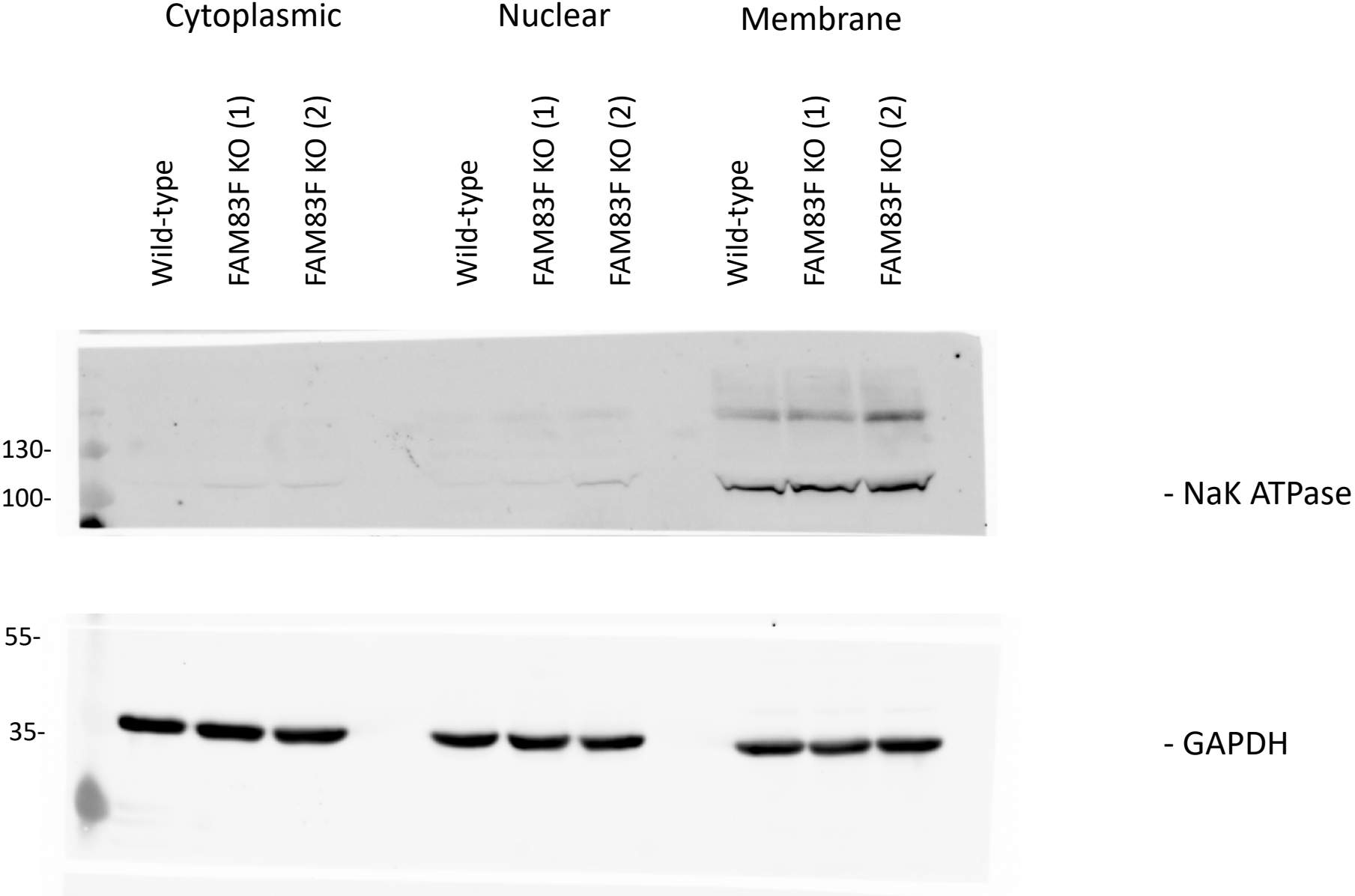

Figure 5B.

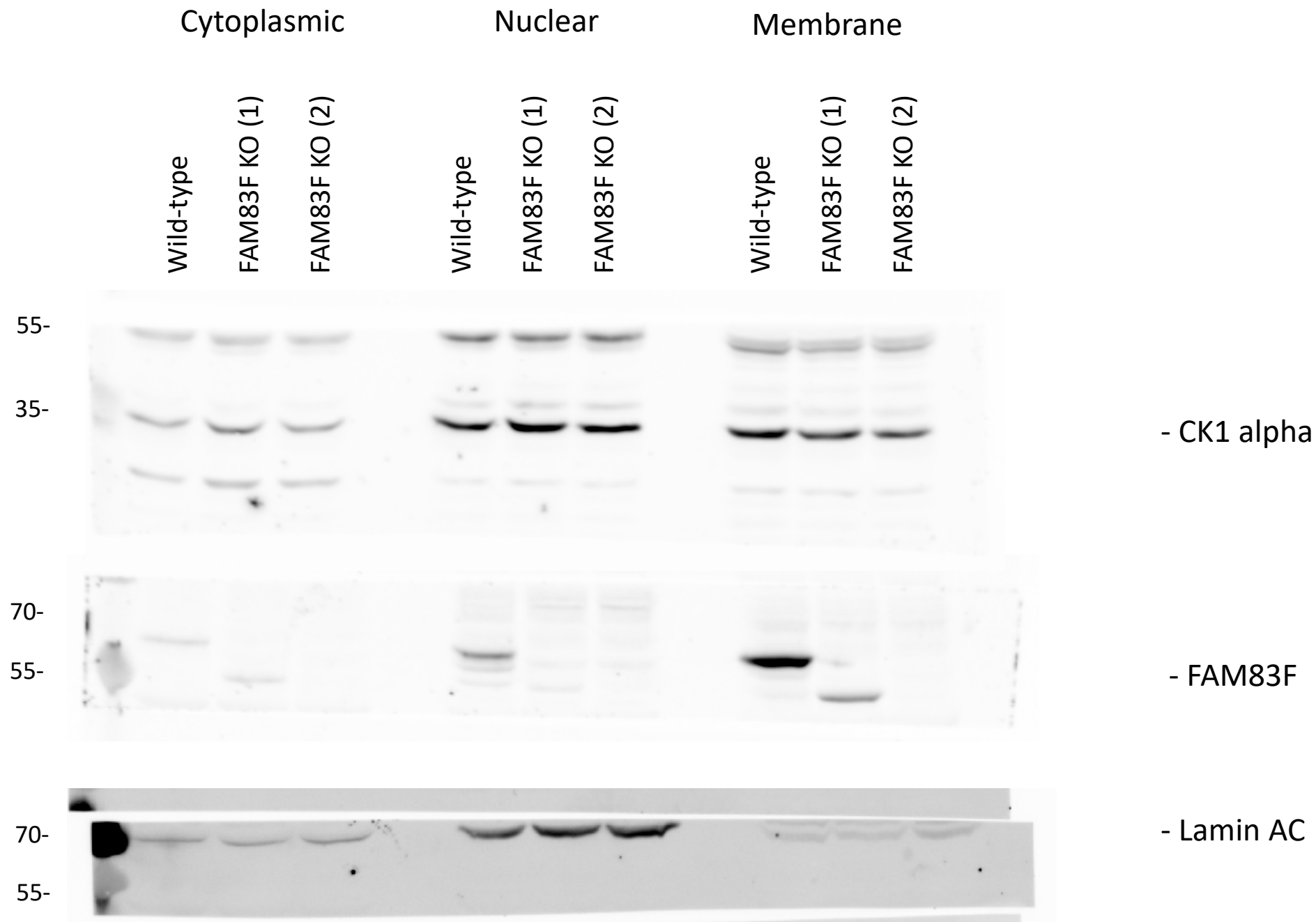

**Figure 5D.**

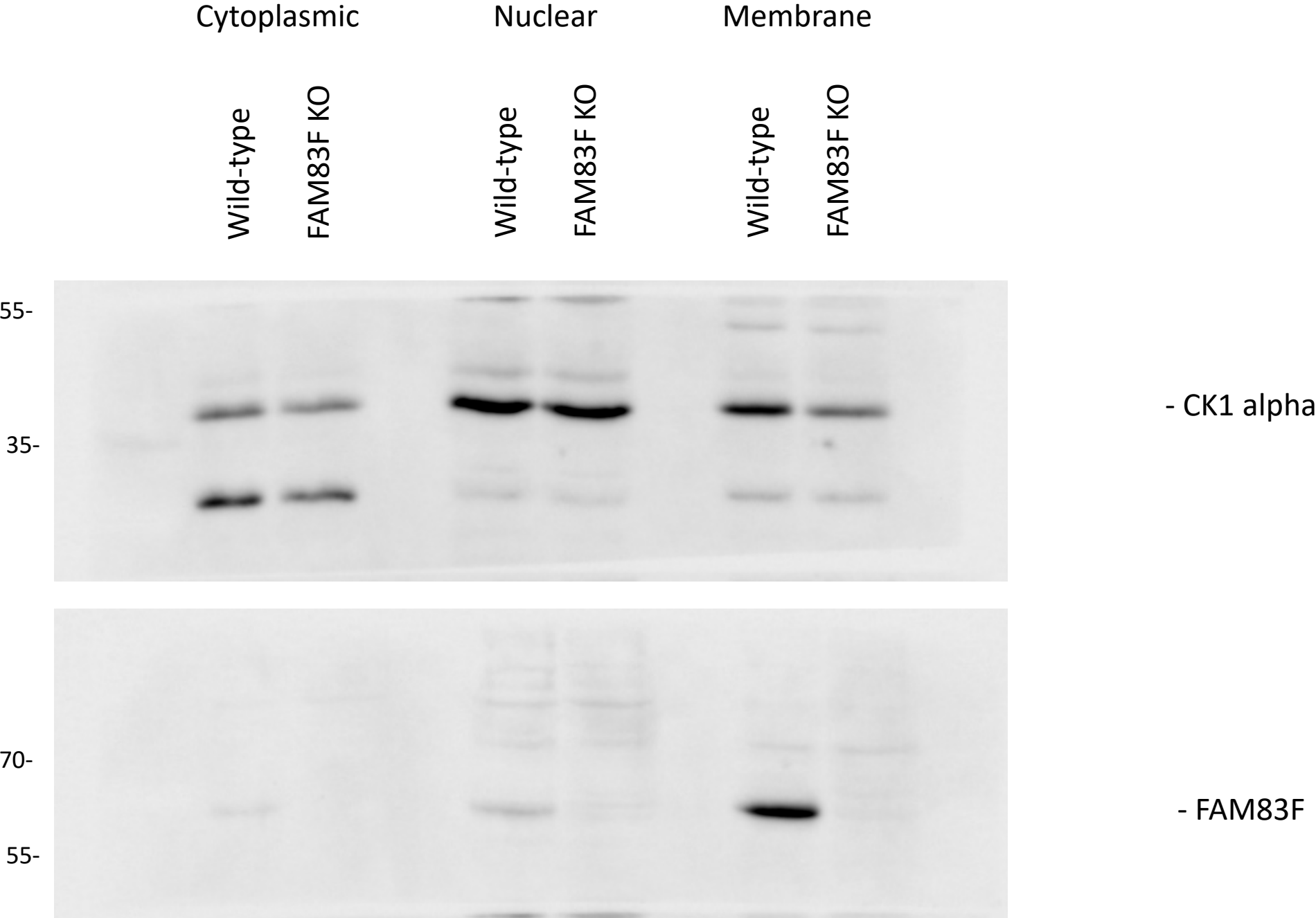

Figure 5D.

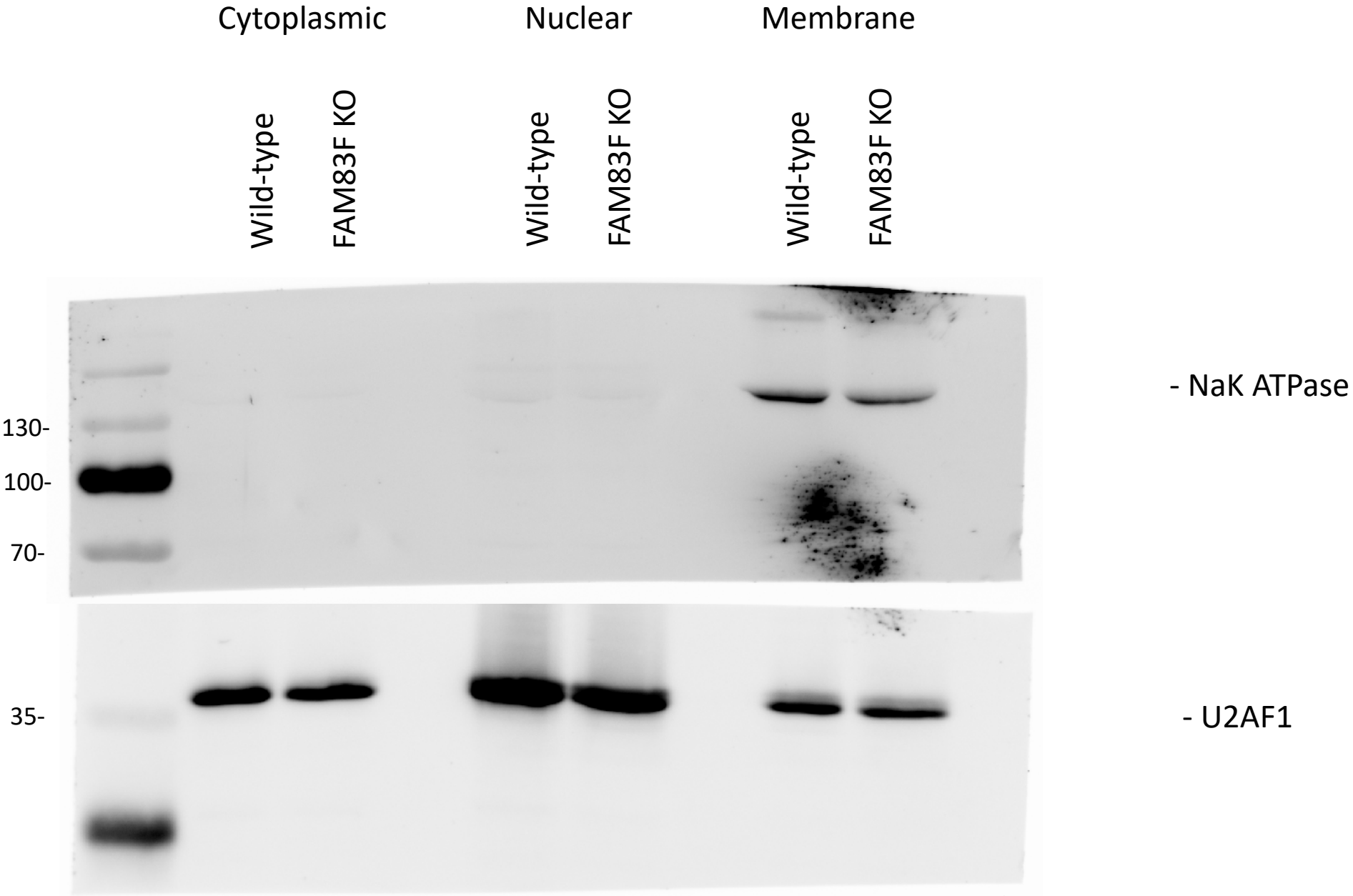

Figure 5D.

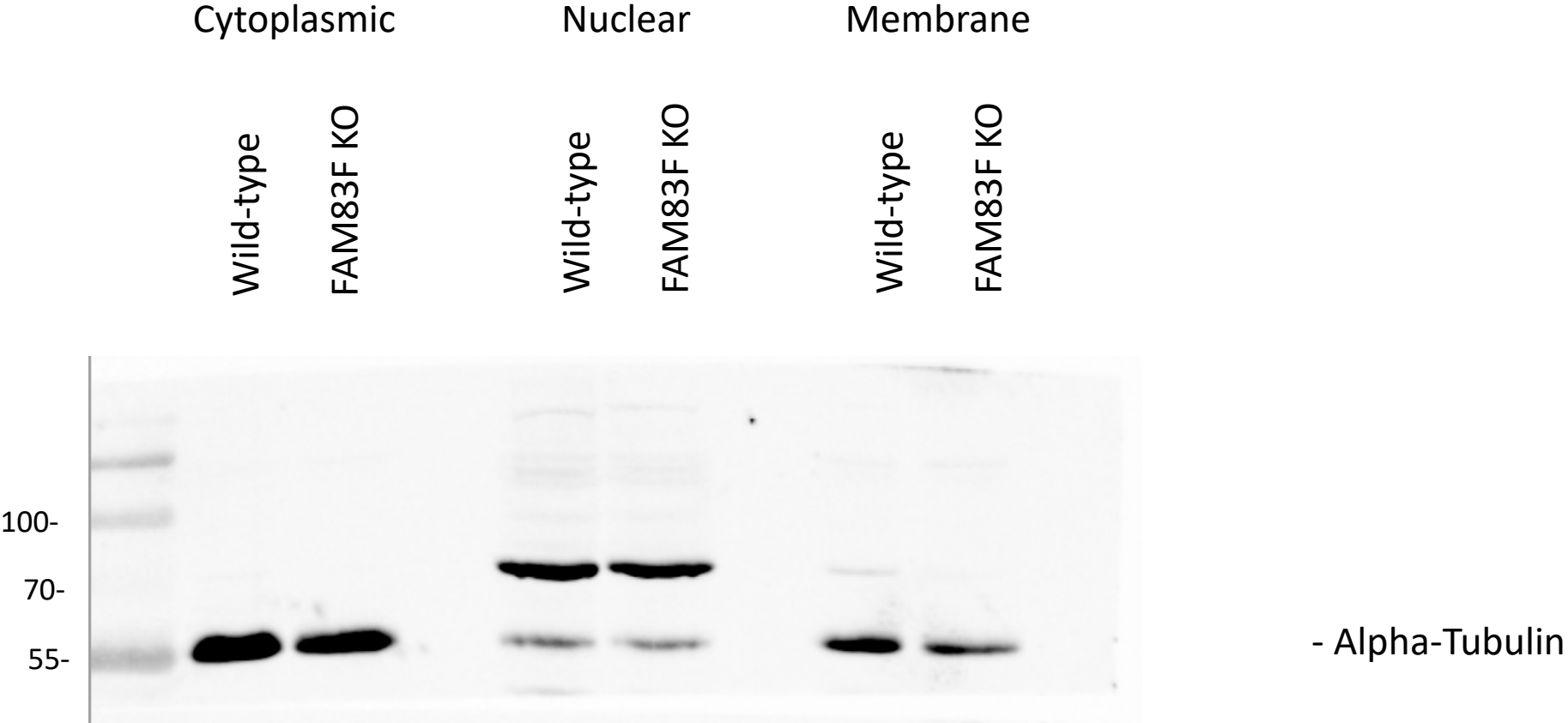

Supplement: Supplementary file 10 [file LSA-2020-00805_SdataF5.pdf]

Figure 6B.

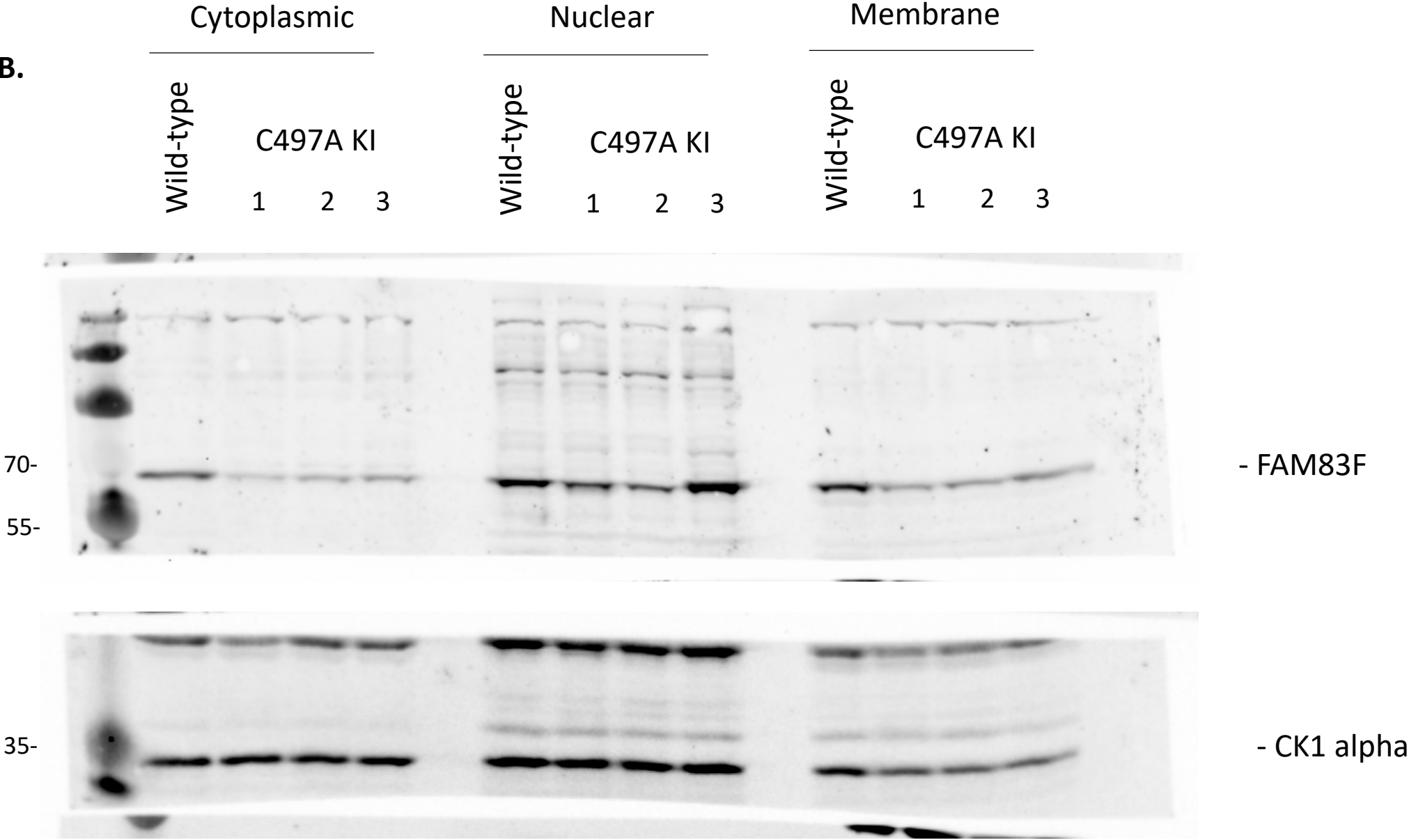

Figure 6B.

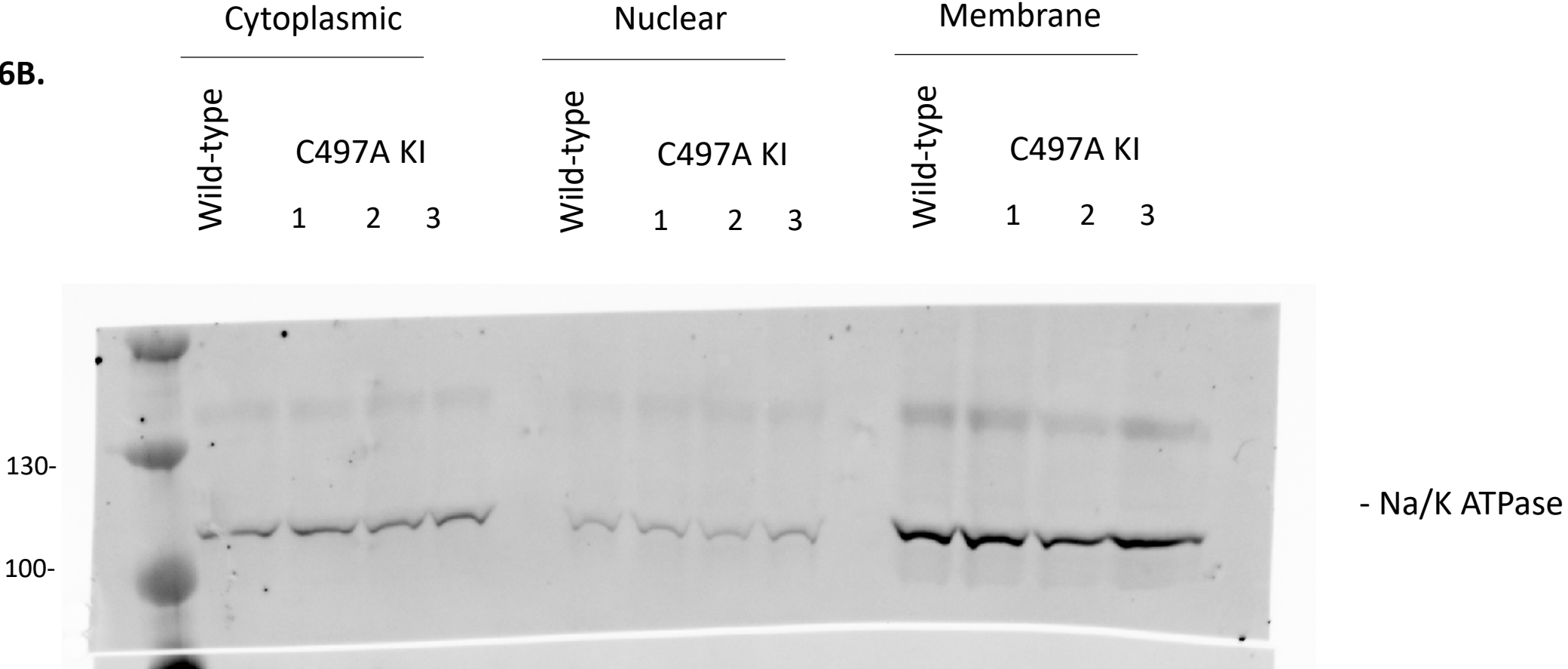

**Figure 6B.**

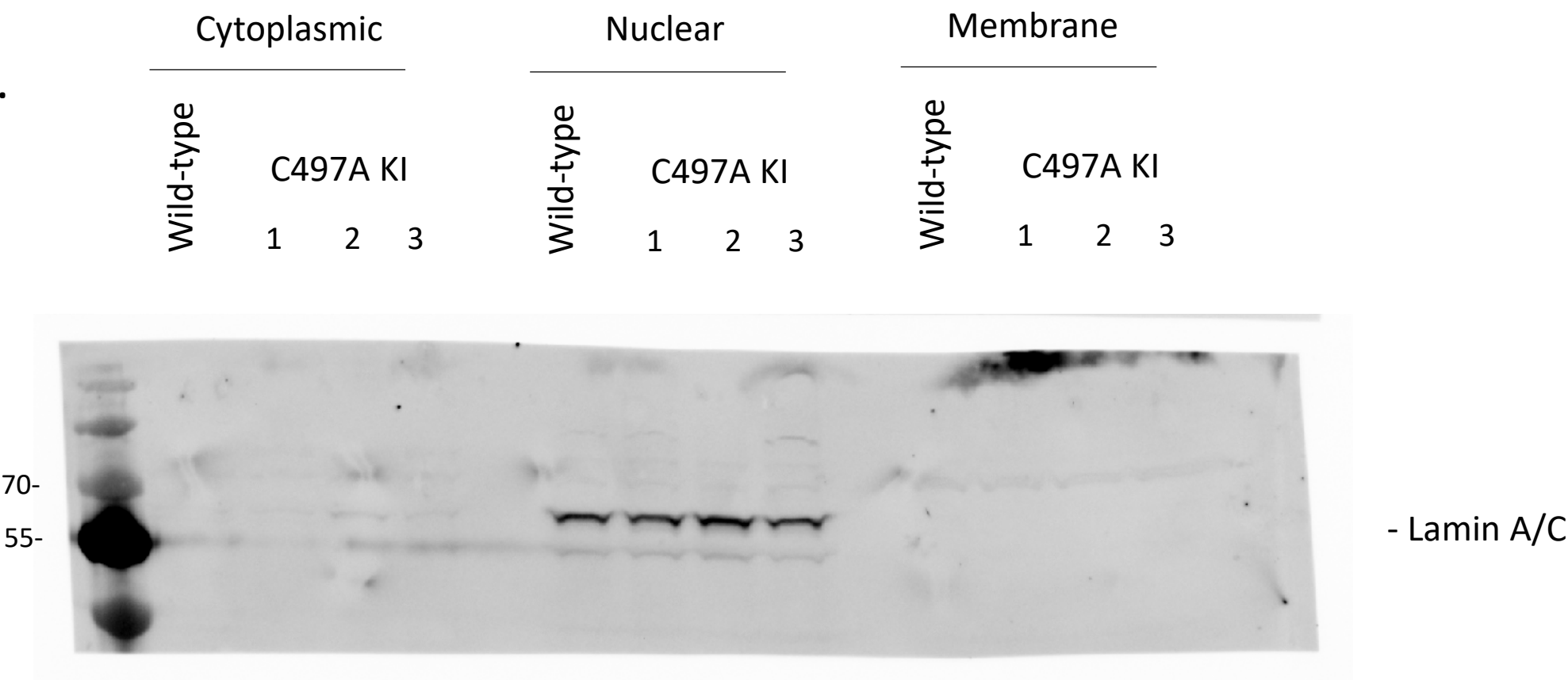

**Figure 6B.**

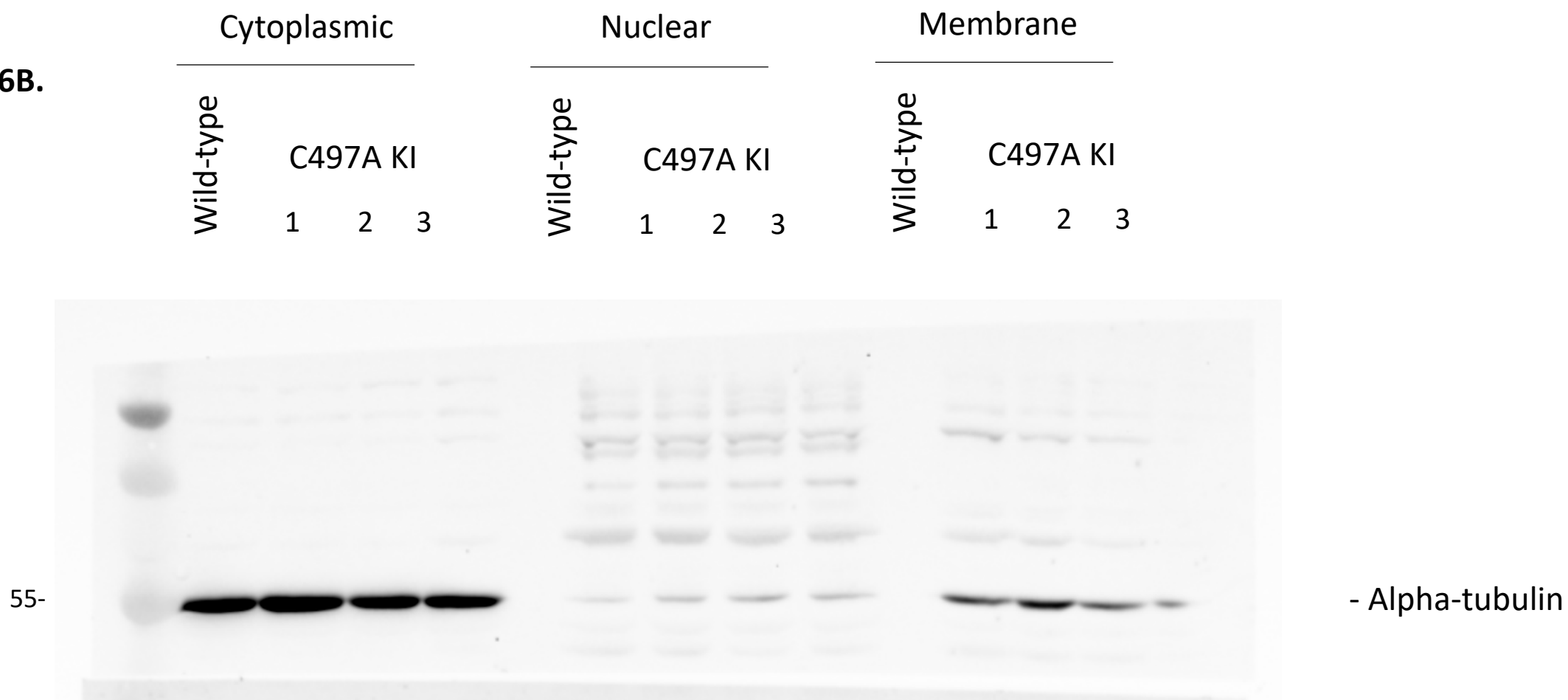

Figure 6D.

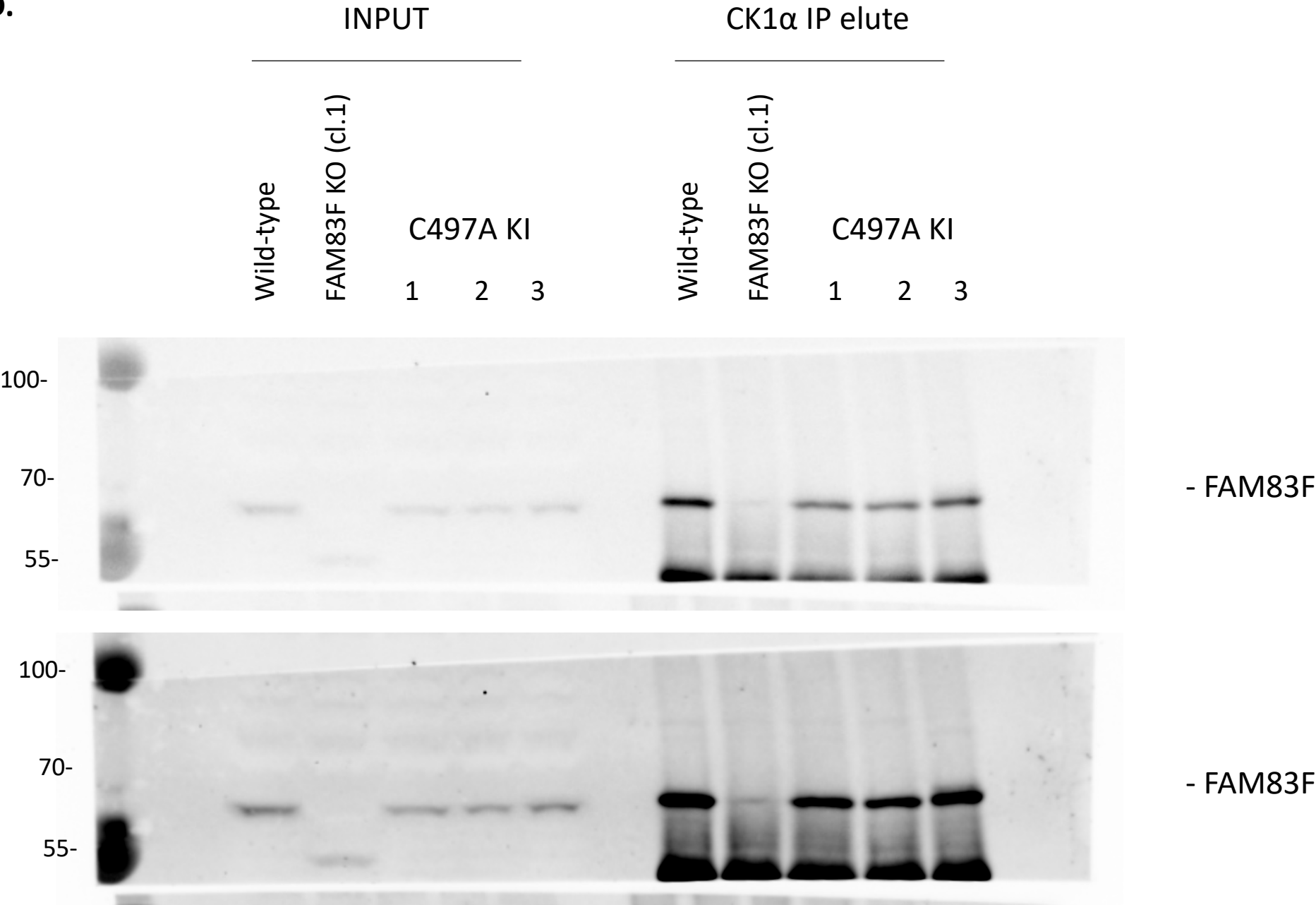

Figure 6D.

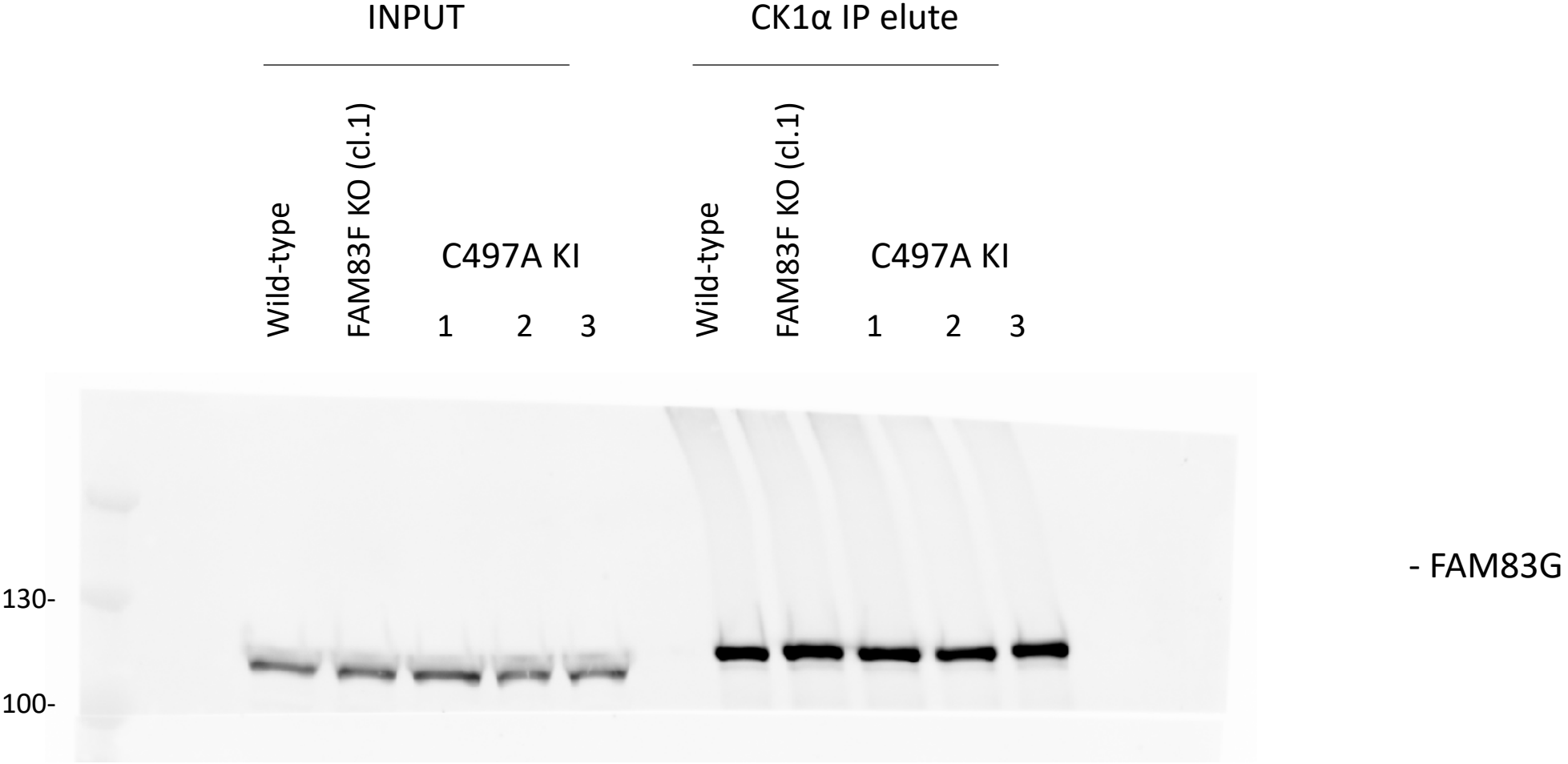

Figure 6D.

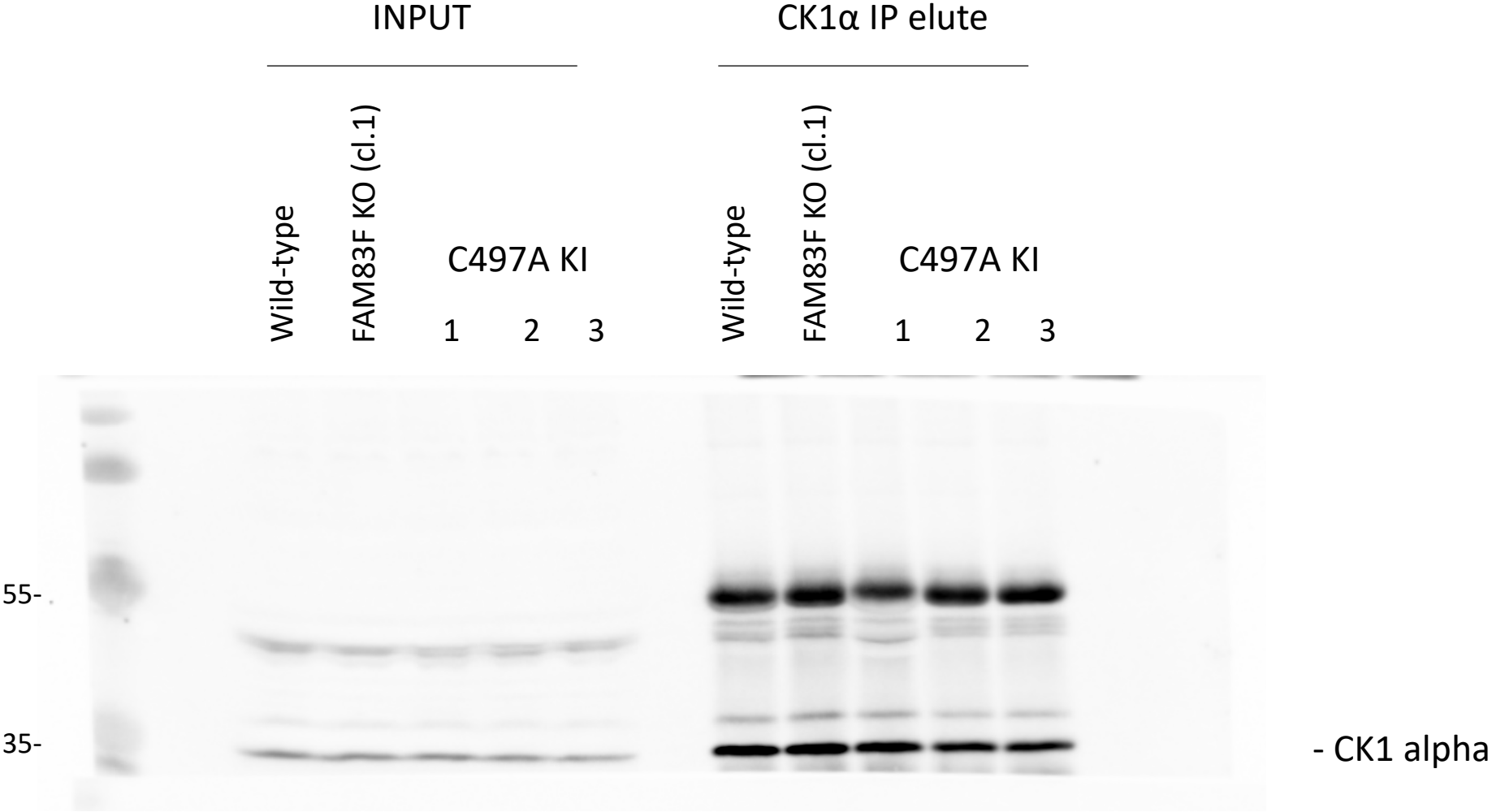

Figure 6D.

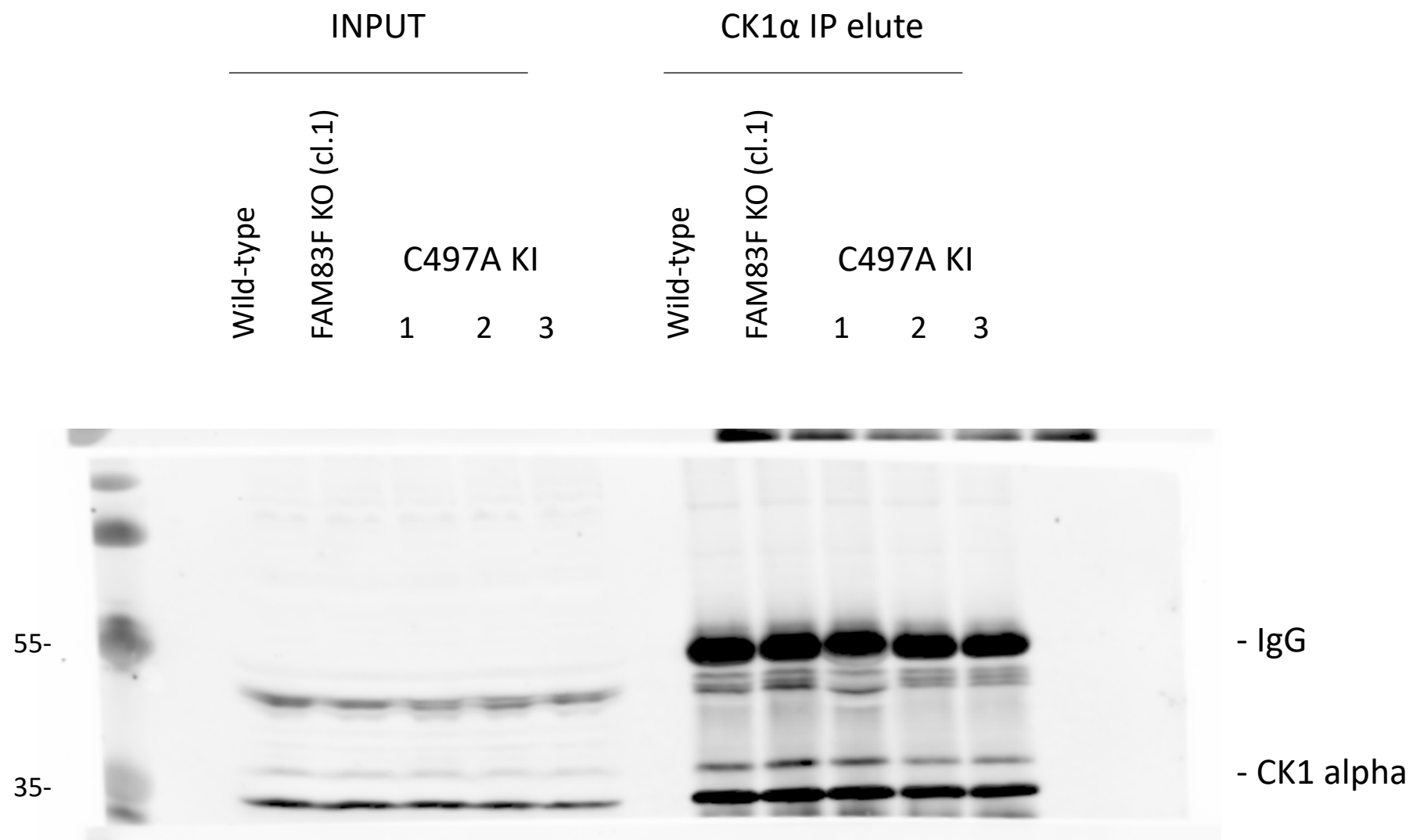

Figure 6D.

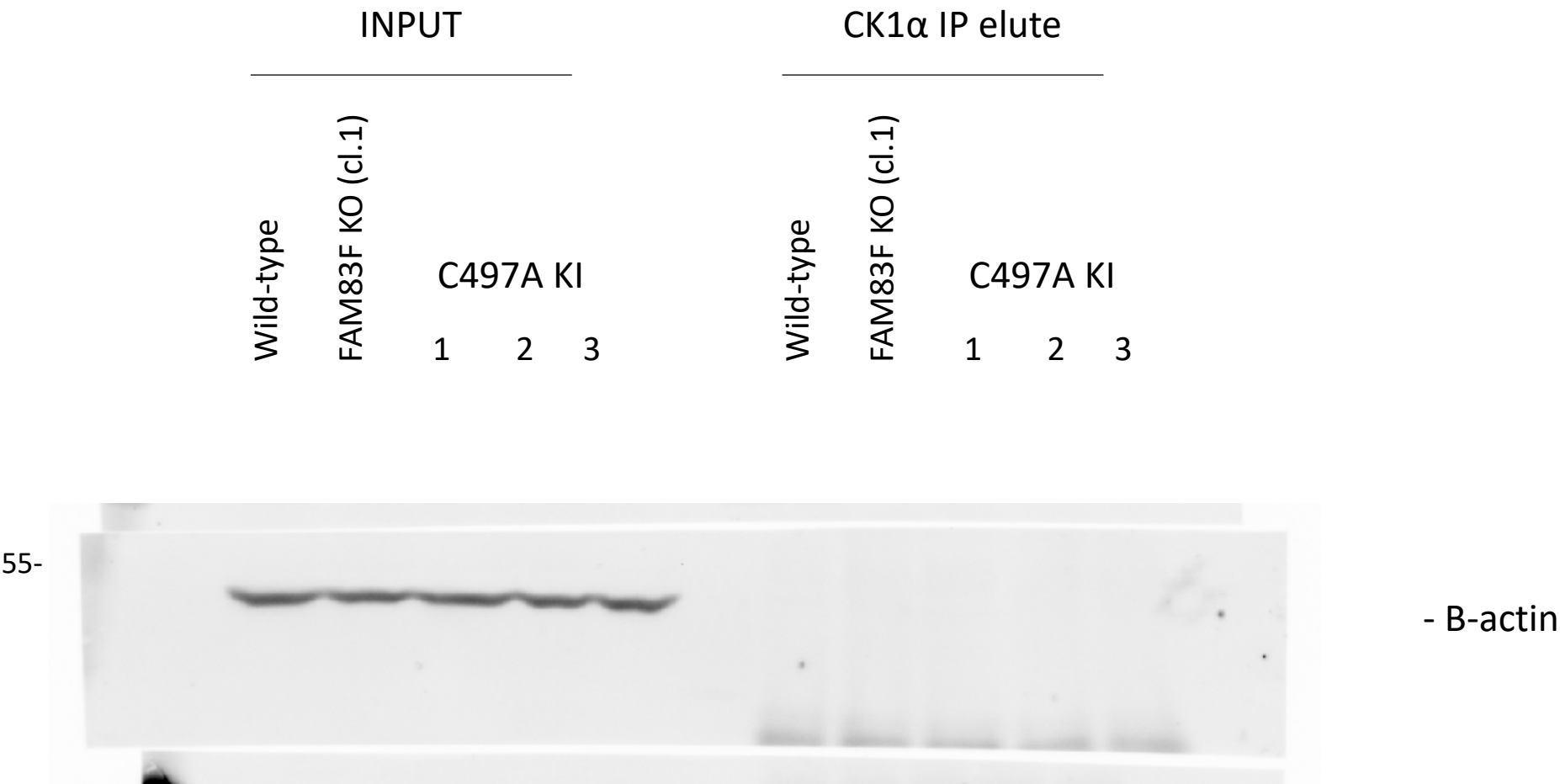

Supplement: Supplementary file 11 [file LSA-2020-00805_SdataF6.pdf]
