# Supplementary material for: FAM83F regulates canonical Wnt signalling through an interaction with CK1α
Source: Life Sci Alliance. 2020 Dec 24;4(2):e202000805. doi: 10.26508/lsa.202000805 (PMC7768192; doi:10.26508/lsa.202000805)

Supplementary Figure 5.

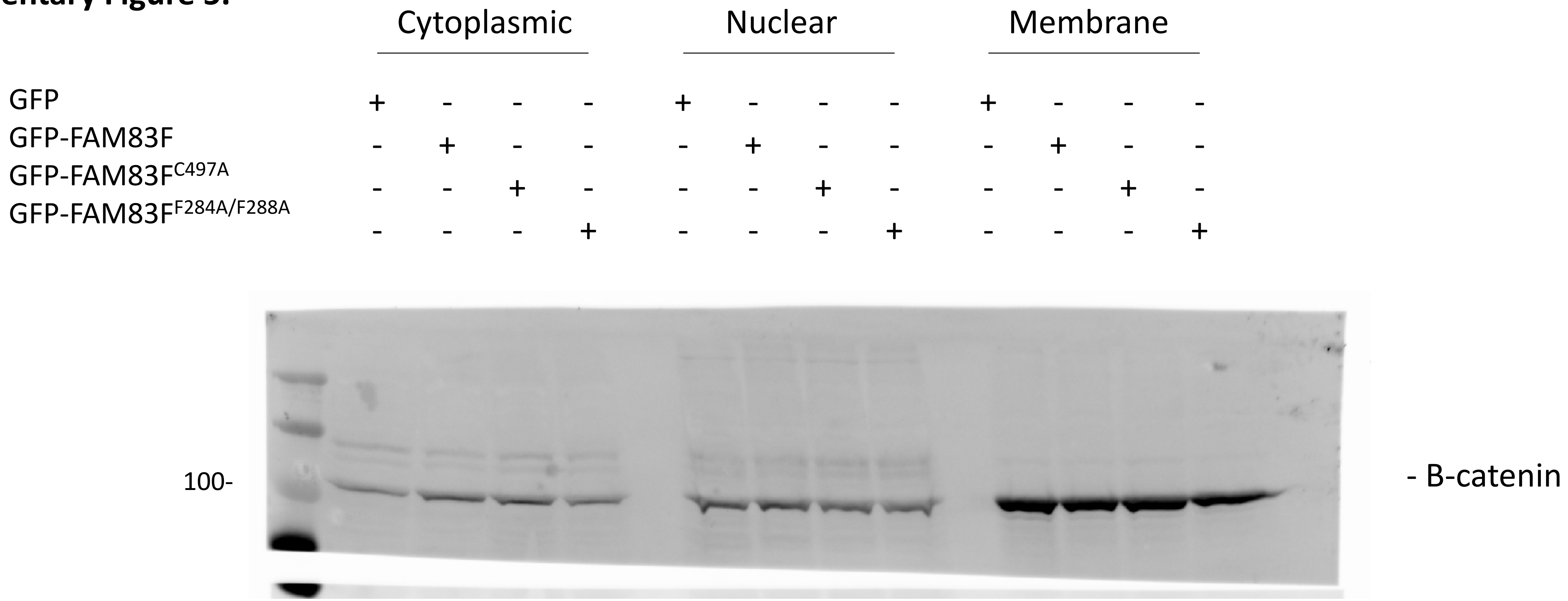

Supplementary Figure 5.

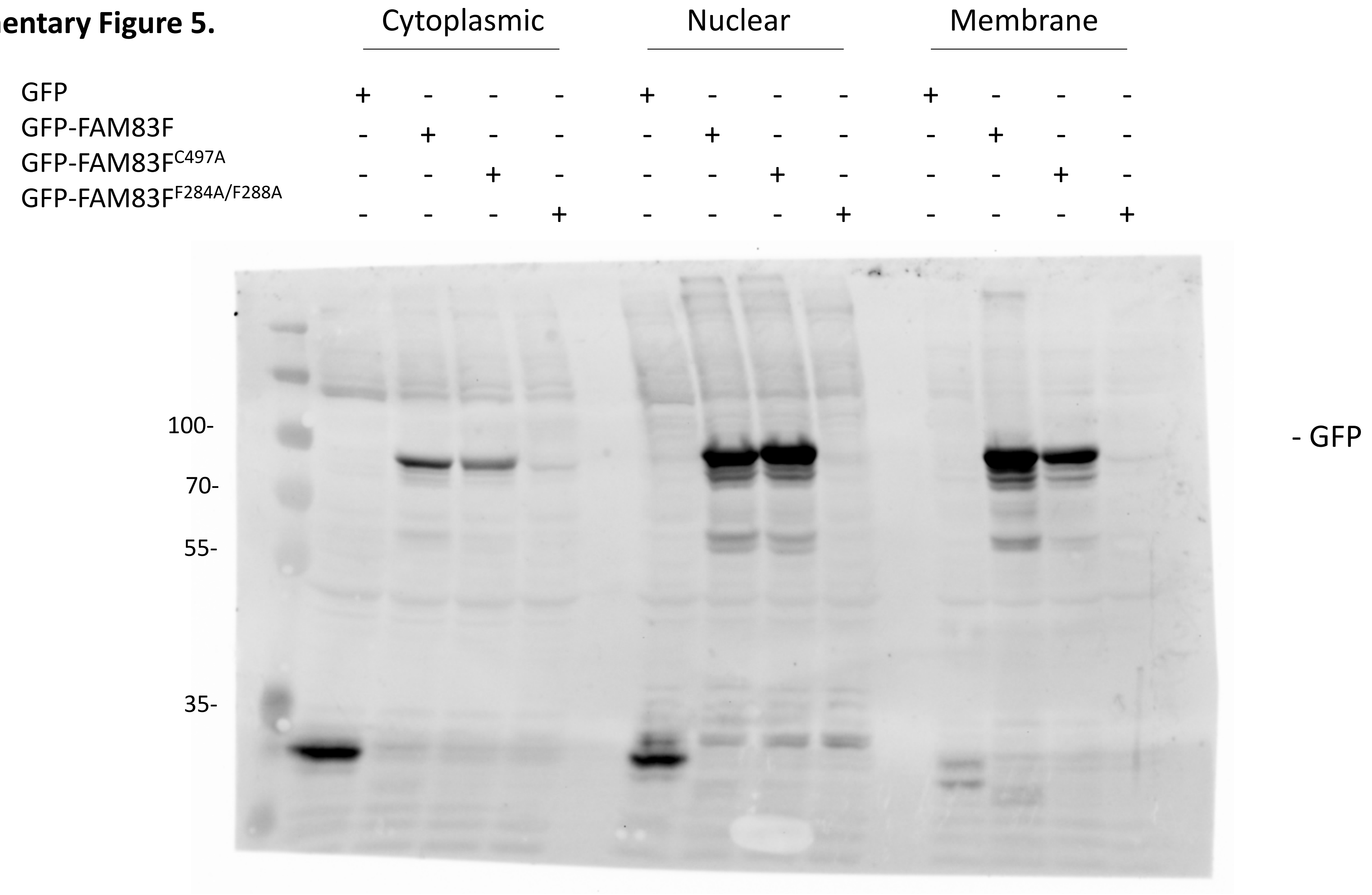

Supplementary Figure 5.

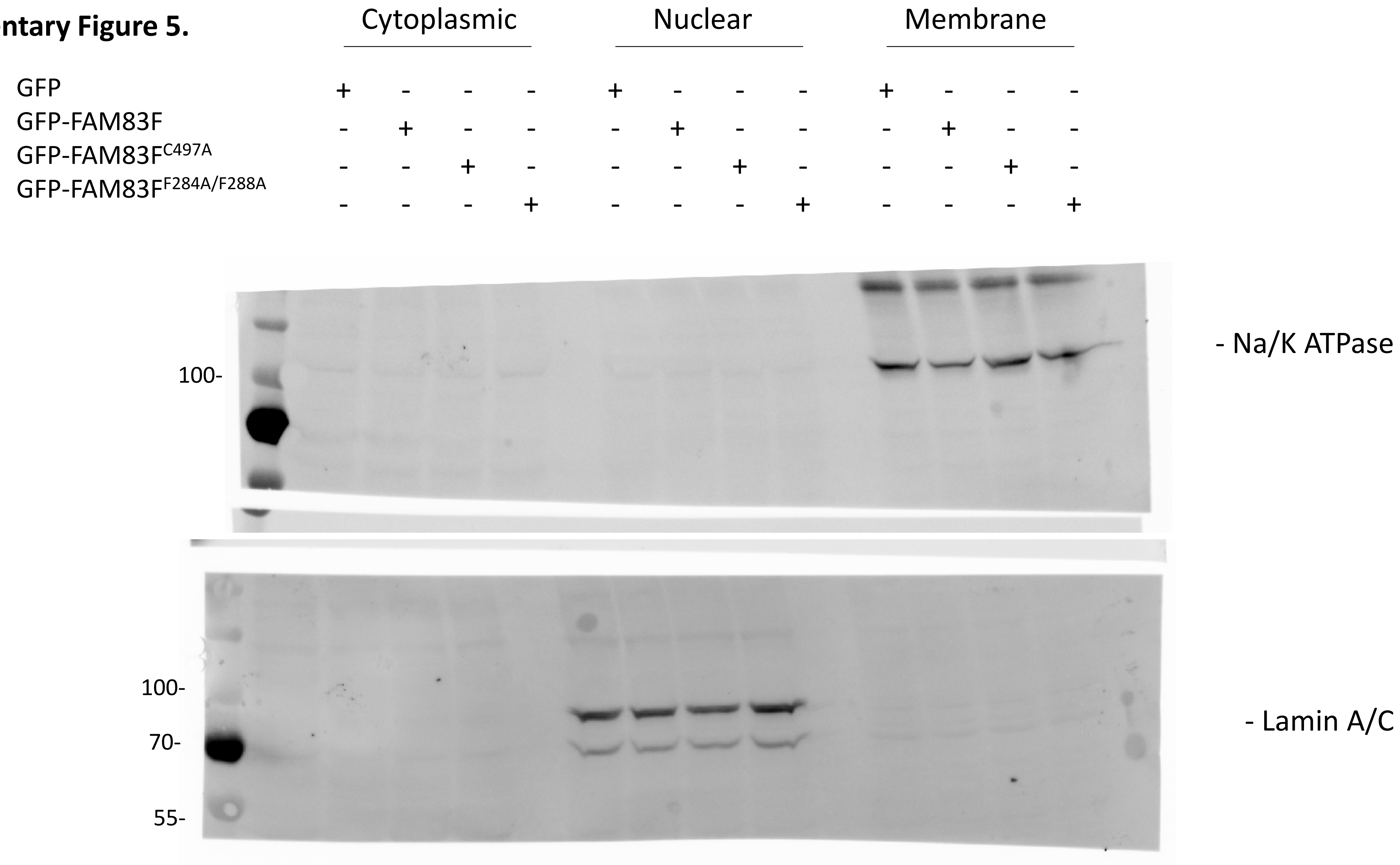

Supplementary Figure 5.

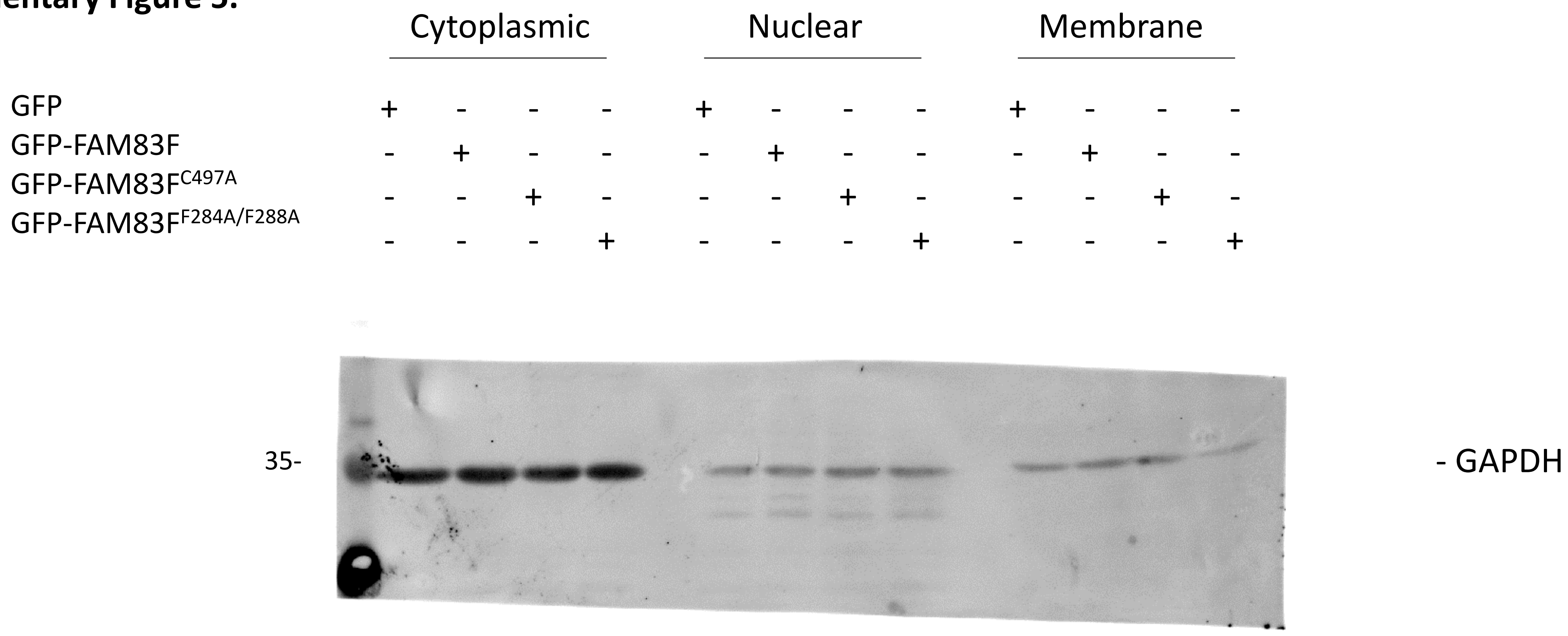

Supplement: Supplementary file 5 [file LSA-2020-00805_SdataFS5.pdf]
