# Supplementary material for: FAM83F regulates canonical Wnt signalling through an interaction with CK1α
Source: Life Sci Alliance. 2020 Dec 24;4(2):e202000805. doi: 10.26508/lsa.202000805 (PMC7768192; doi:10.26508/lsa.202000805)

**Figure 4B.**

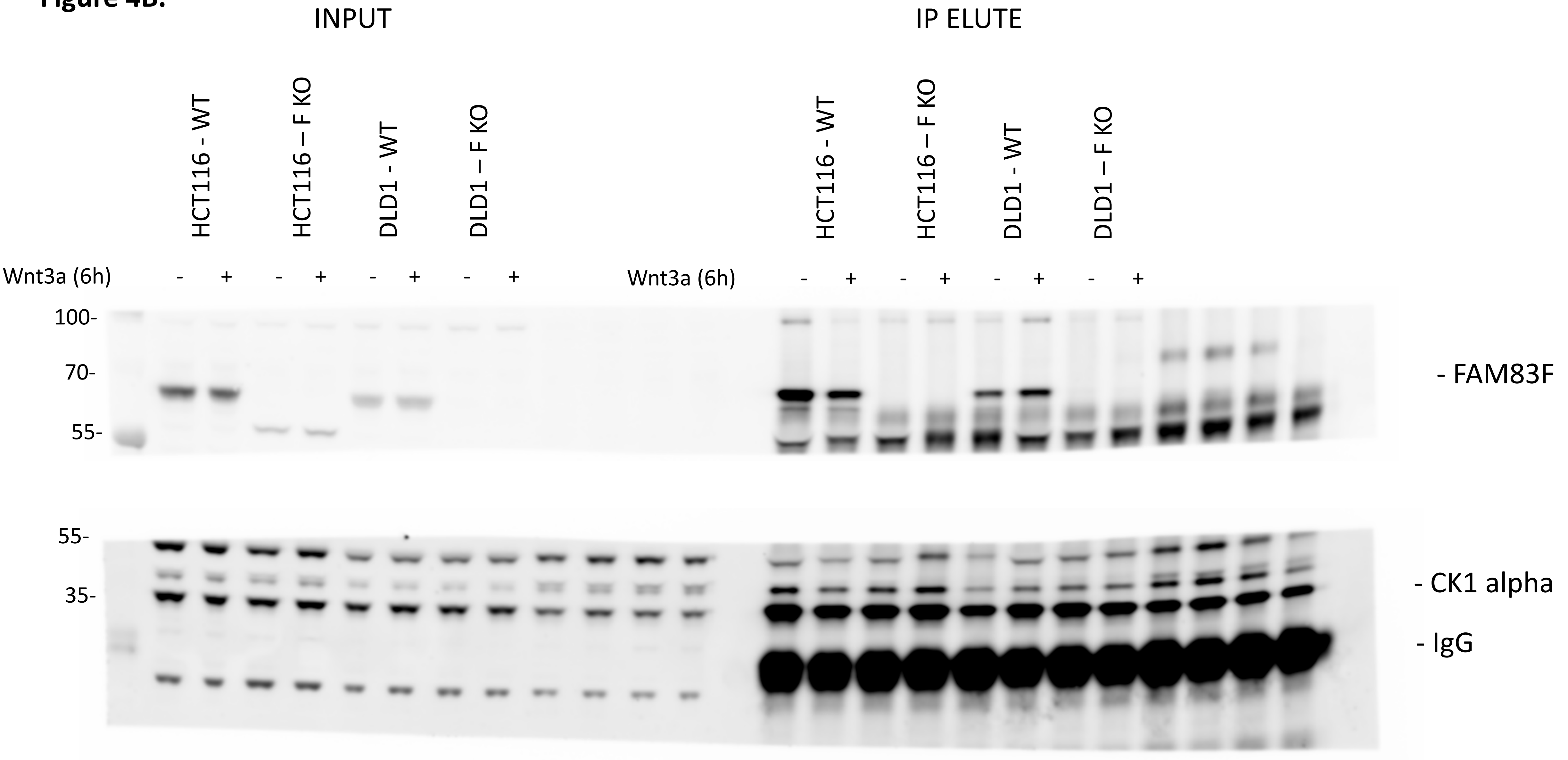

INPUT
IP ELUTE
HCT116 - WT
HCT116 – F KO
DLD1 - WT
DLD1 – F KO
Wnt3a (6h)
- + - + - + - +
100-
70-
55-
35-
- FAM83F
- CK1 alpha
- IgG

**Figure 4B.**

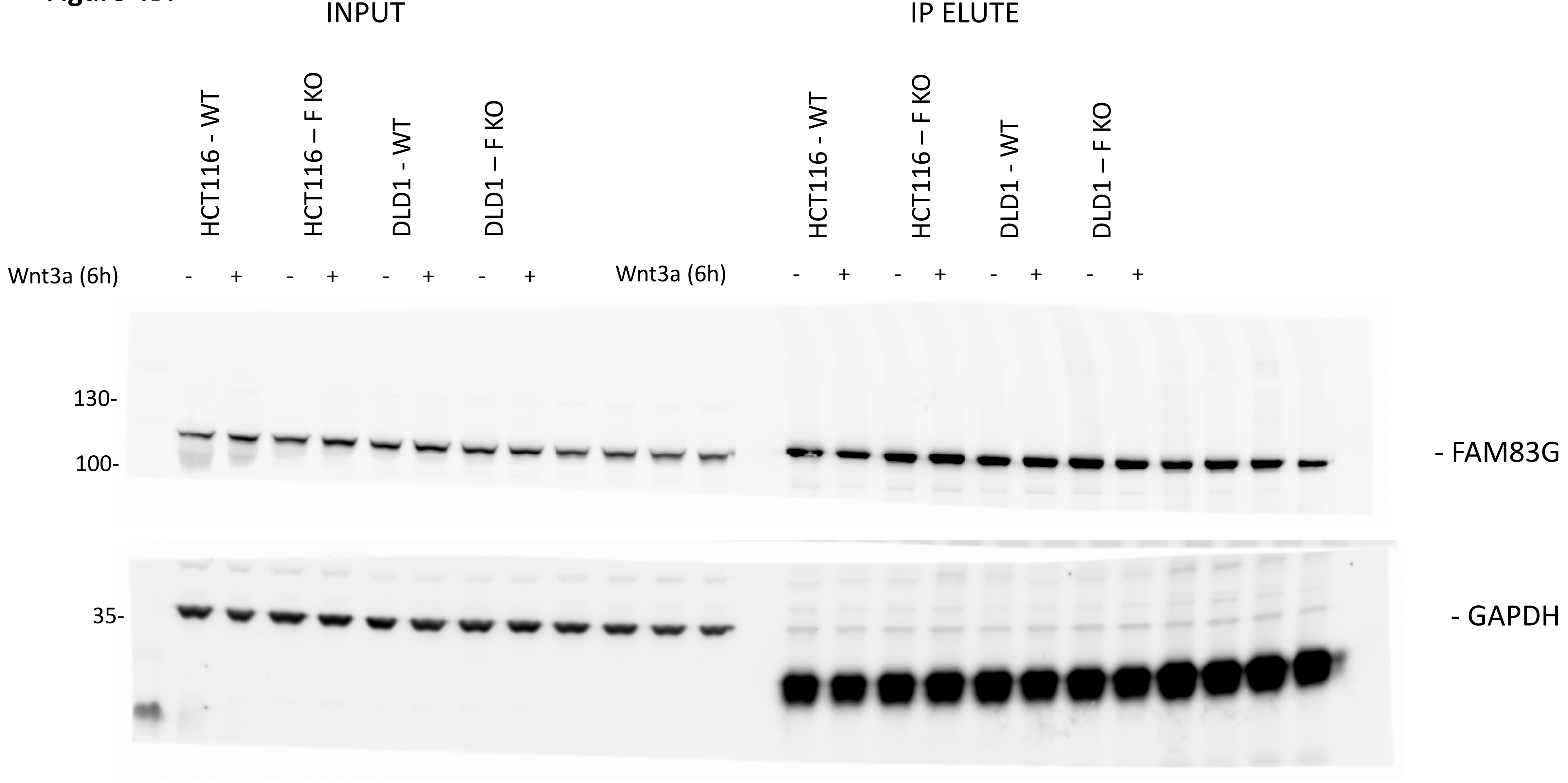
INPUT
IP ELUTE
HCT116 - WT
HCT116 – F KO
DLD1 - WT
DLD1 – F KO
HCT116 - WT
HCT116 – F KO
DLD1 - WT
DLD1 – F KO
Wnt3a (6h)
- + - + - + - +
Wnt3a (6h)
- + - + - + - +
130-
100-
- FAM83G
35-
- GAPDH

**Figure 4C.**

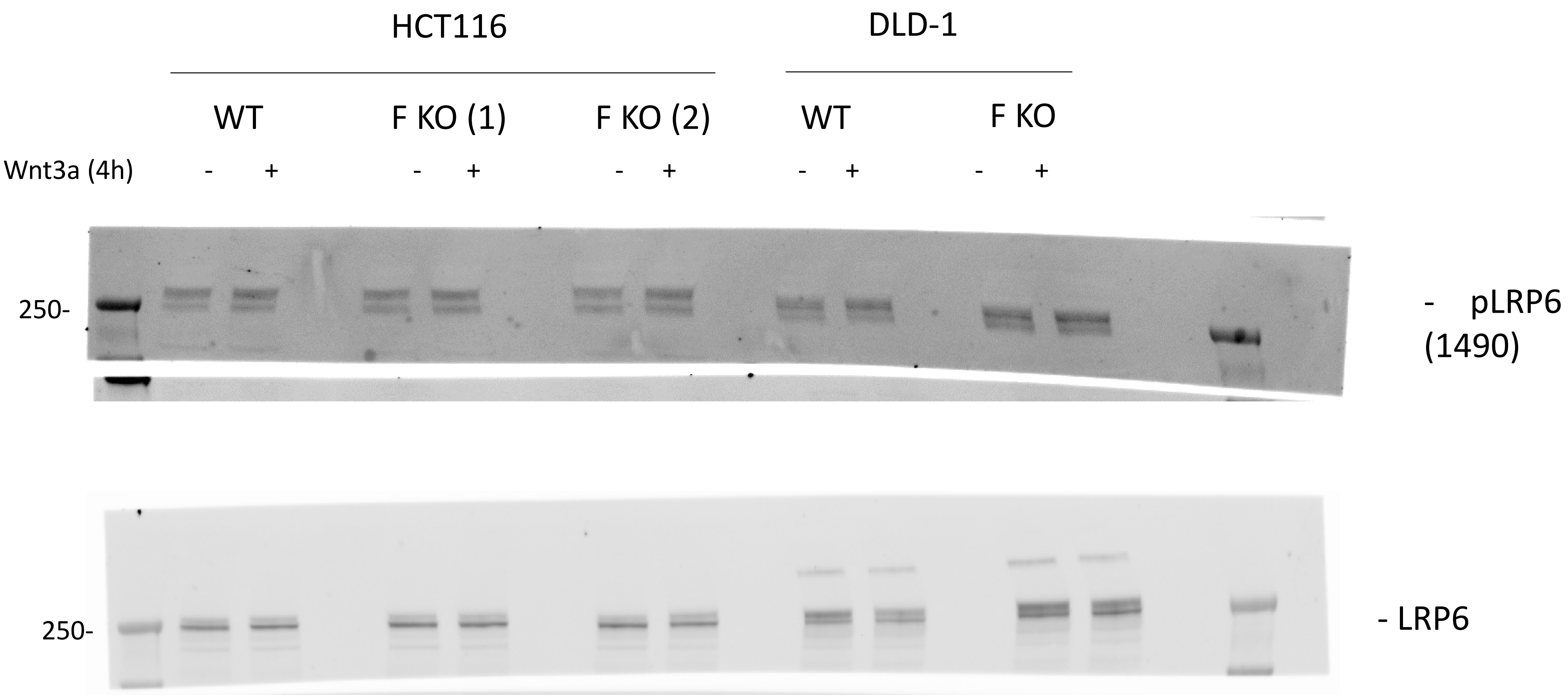
HCT116
DLD-1
WT
F KO (1)
F KO (2)
WT
F KO
Wnt3a (4h)
- + - + - + - + - +
250-
- pLRP6 (1490)
250-
- LRP6

**Figure 4C.**

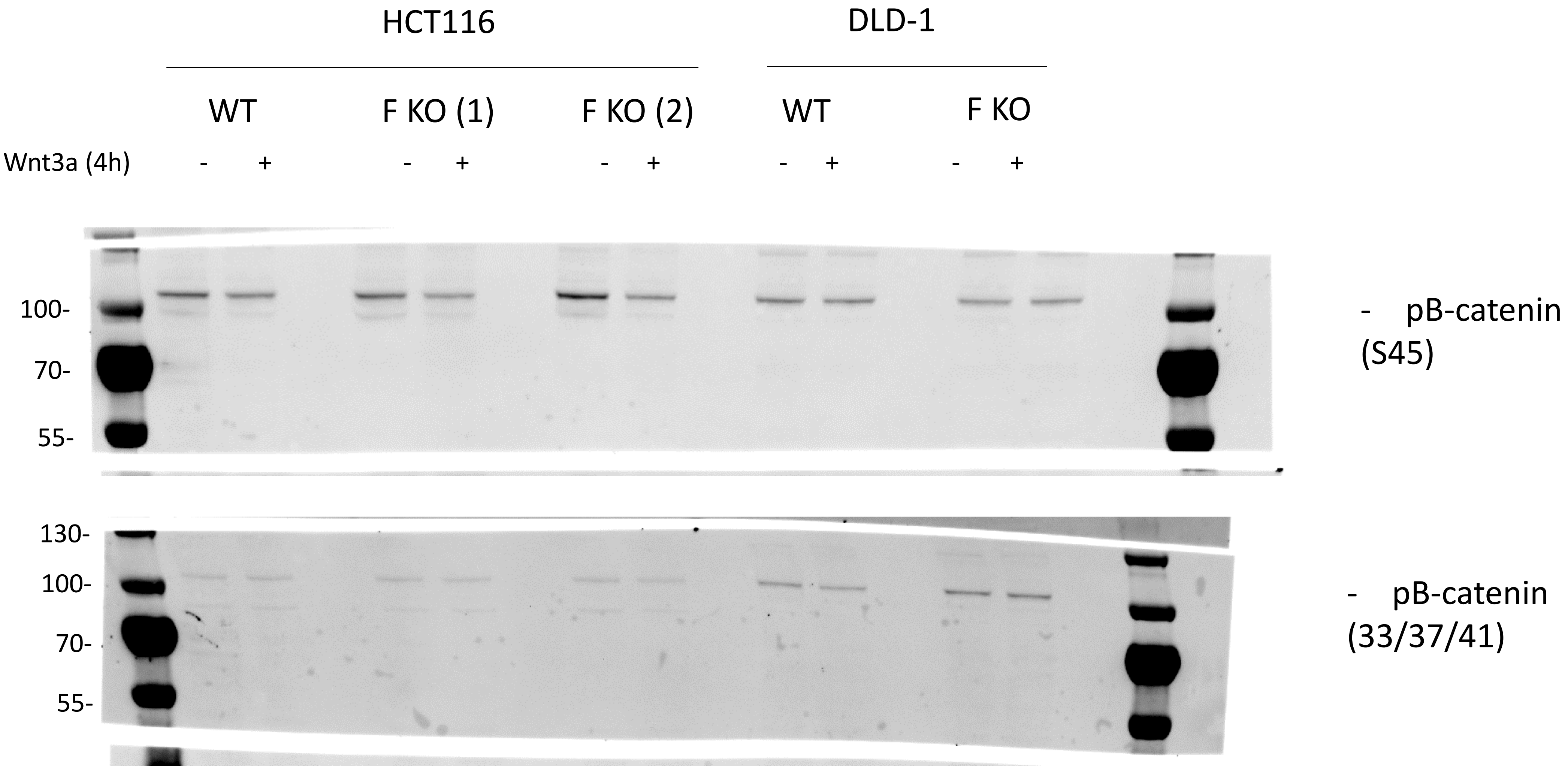
HCT116
DLD-1
WT
F KO (1)
F KO (2)
WT
F KO
Wnt3a (4h)
- +
- +
- +
- +
- +
100-
70-
55-
- pB-catenin (S45)
130-
100-
70-
55-
- pB-catenin (33/37/41)

**Figure 4C.**

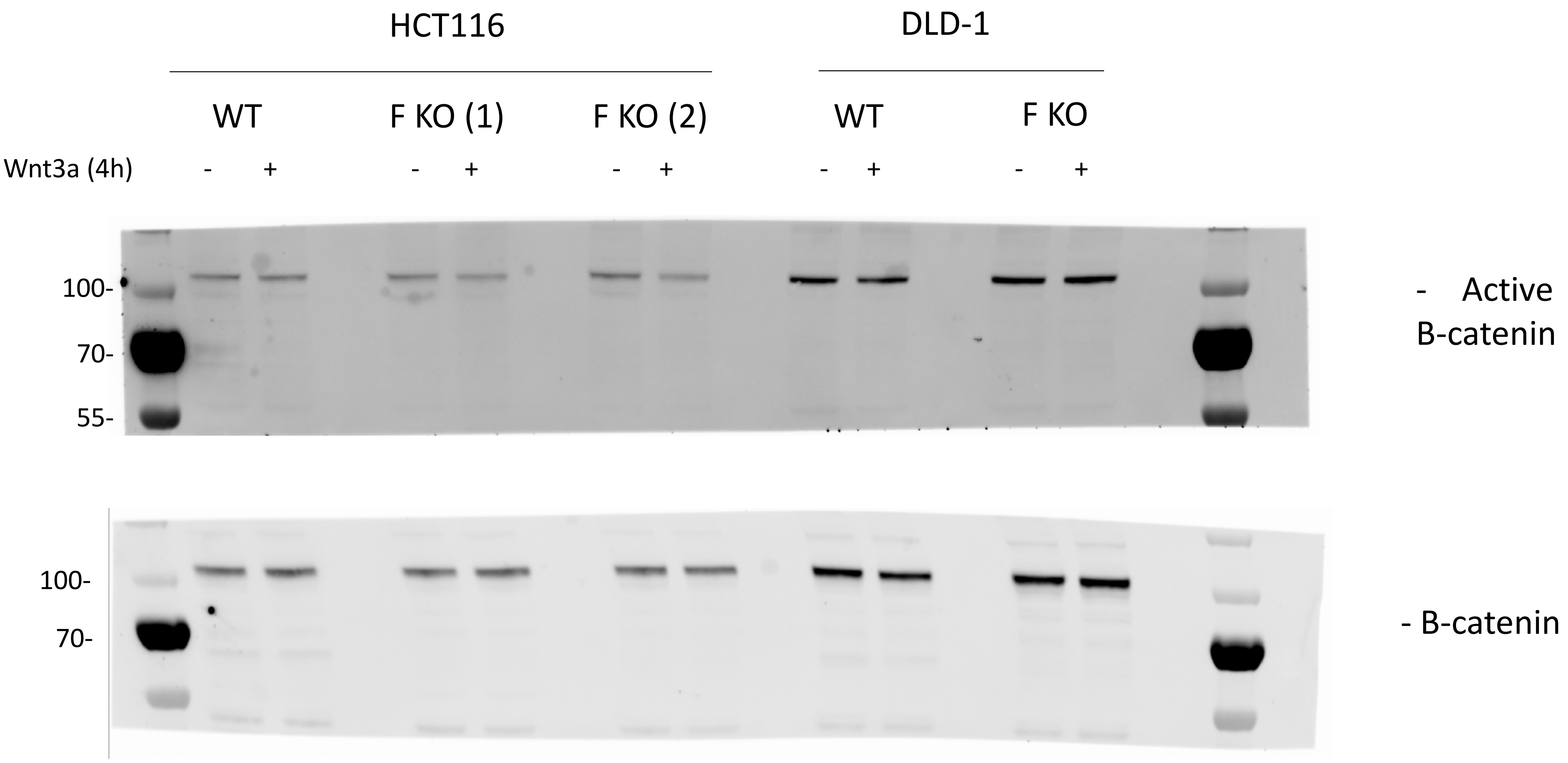
HCT116
DLD-1
WT
F KO (1)
F KO (2)
WT
F KO
Wnt3a (4h)
- + - + - + - + - +
100-
70-
55-
- Active B-catenin
100-
70-
- B-catenin

**Figure 4C.**

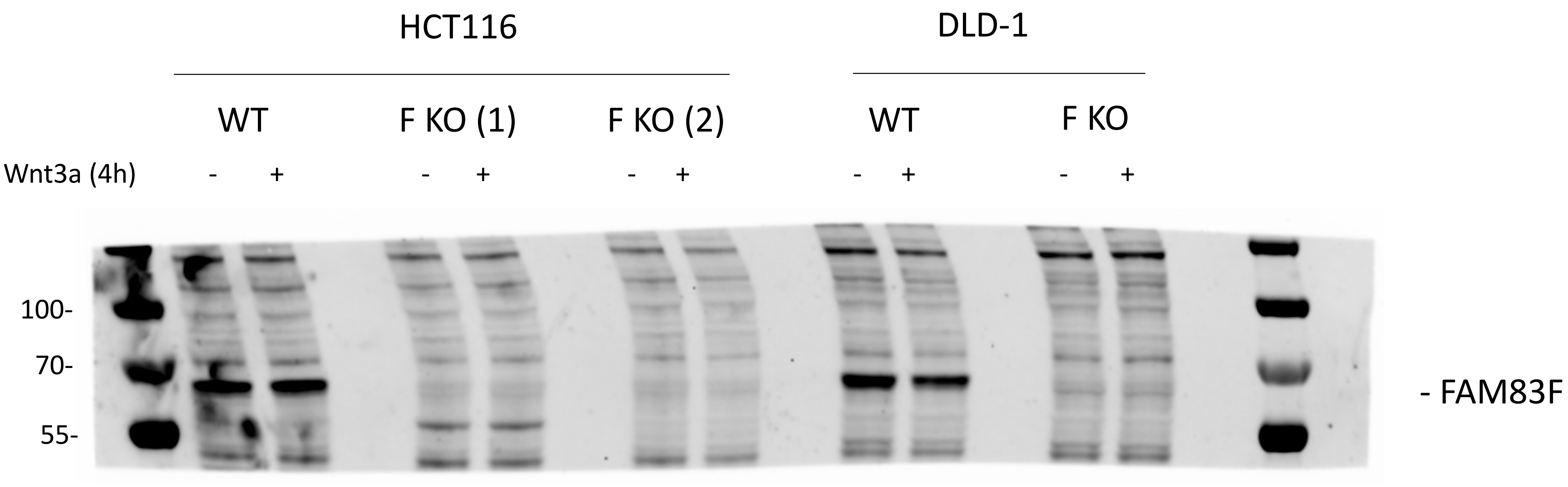

**Figure 4C.**

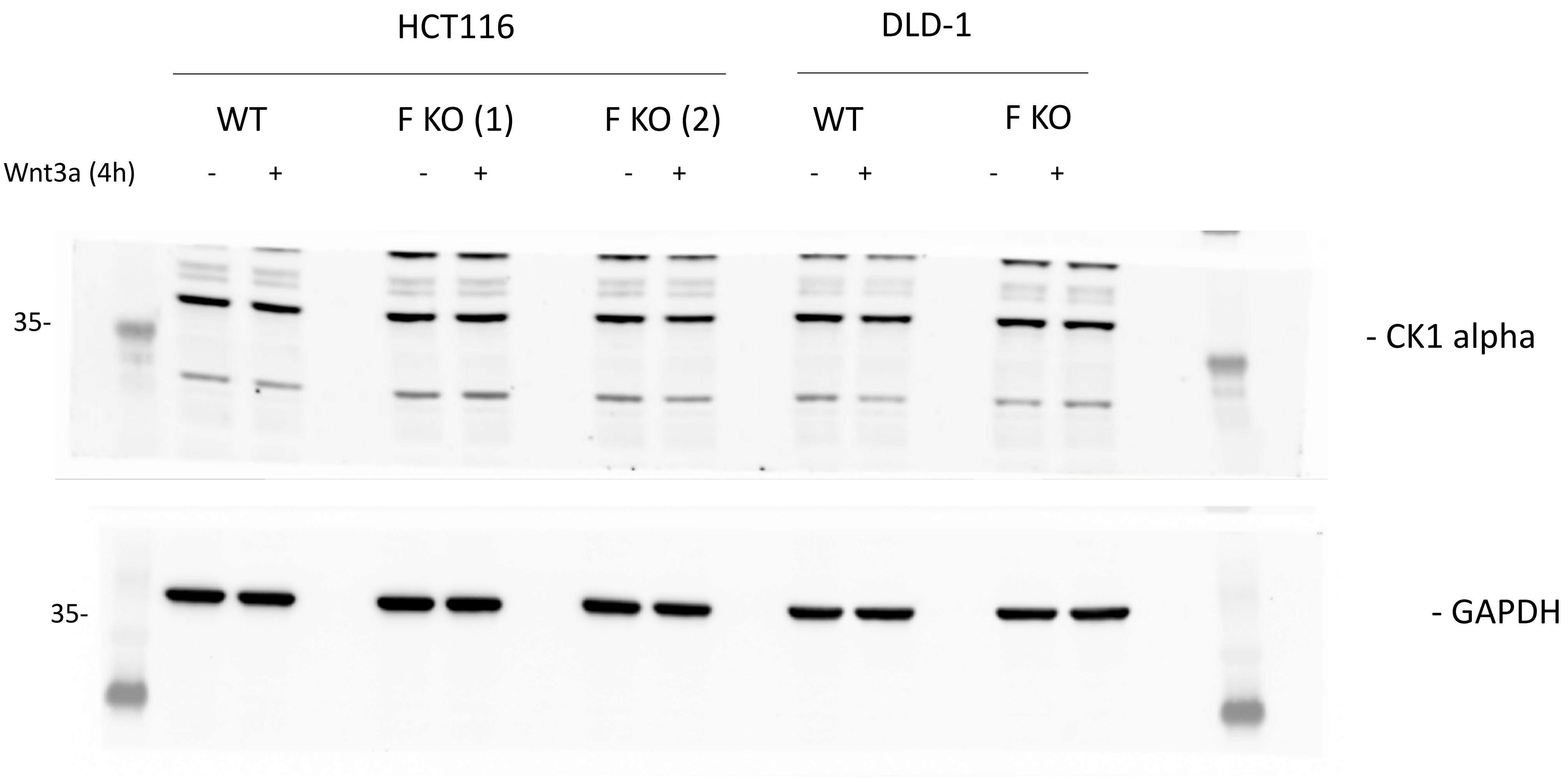

Supplement: Supplementary file 8 [file LSA-2020-00805_SdataF4.pdf]
