# Supplementary material for: FAM83F regulates canonical Wnt signalling through an interaction with CK1α
Source: Life Sci Alliance. 2020 Dec 24;4(2):e202000805. doi: 10.26508/lsa.202000805 (PMC7768192; doi:10.26508/lsa.202000805)

Supplementary Figure 11A.

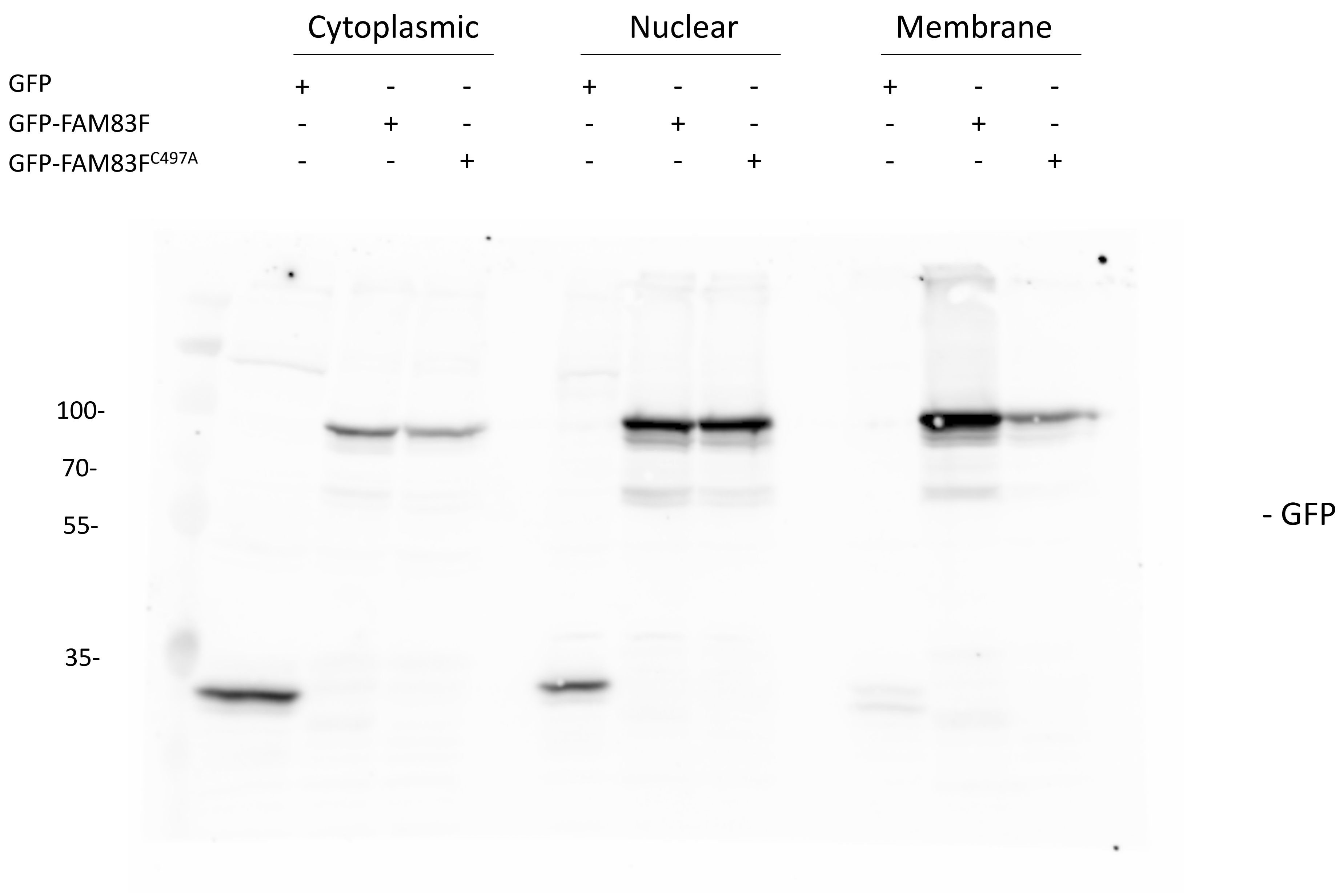

Supplementary Figure 11A.

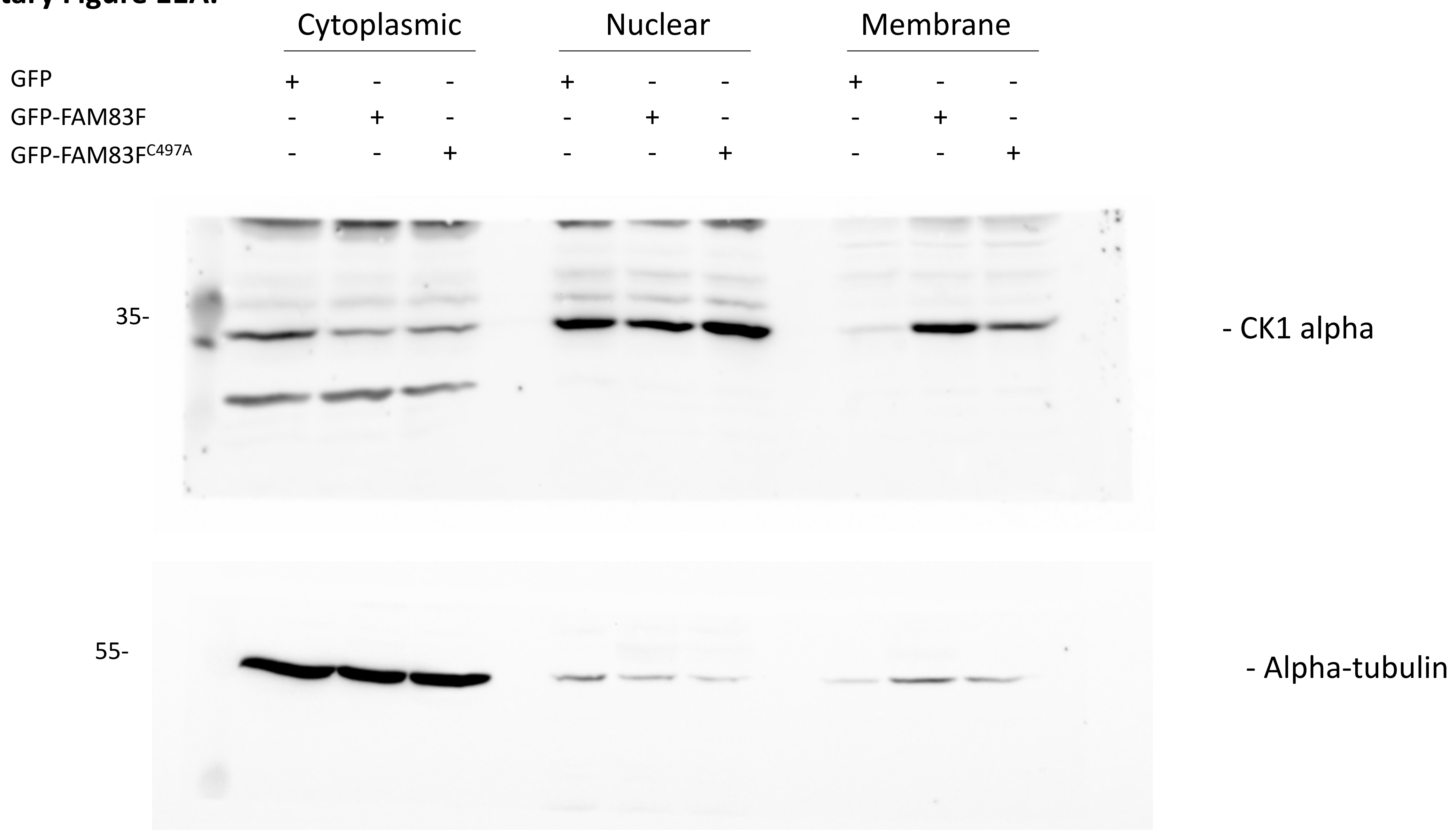

Supplementary Figure 11A.

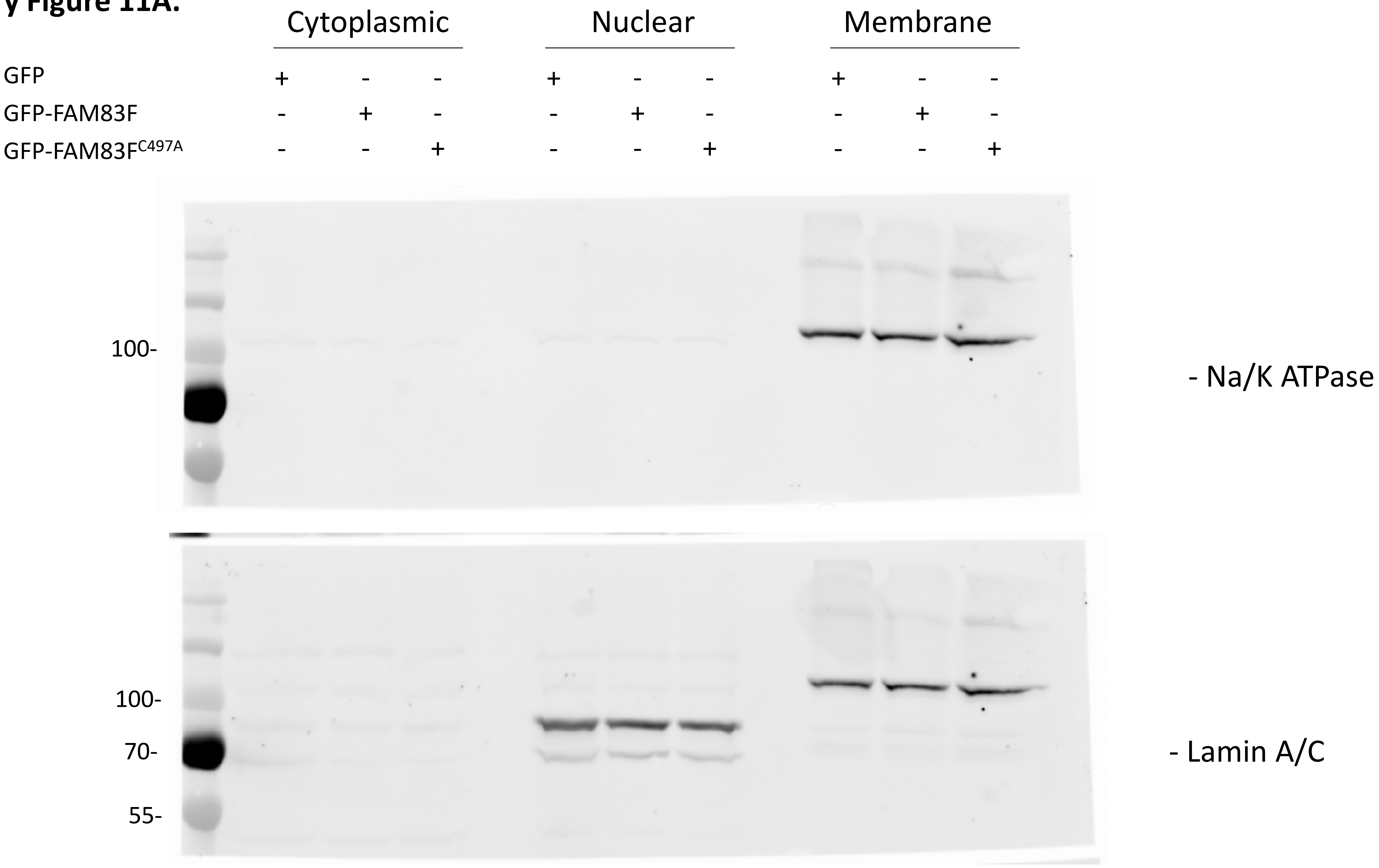

Supplementary Figure 11B.

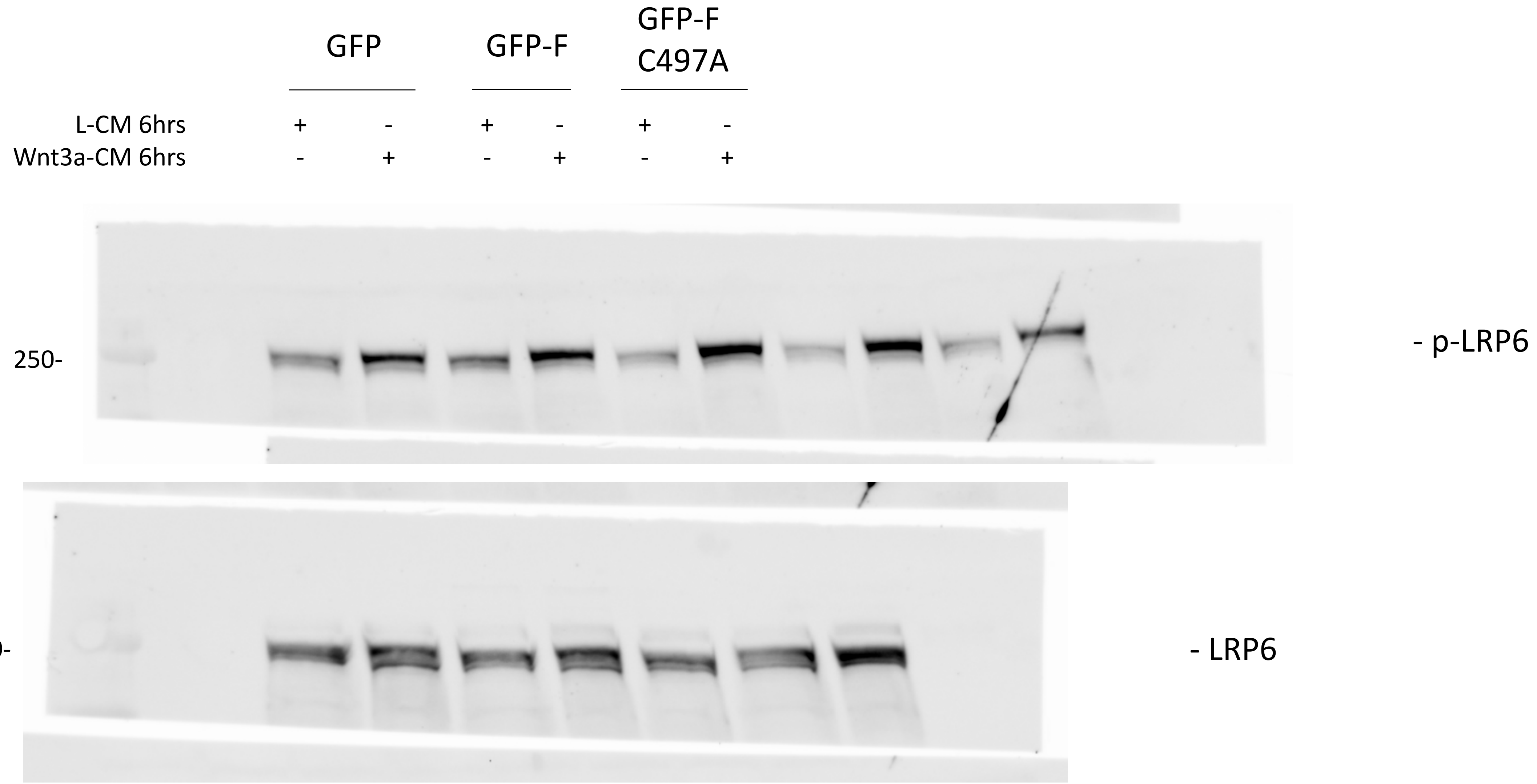

Supplementary Figure 11B.

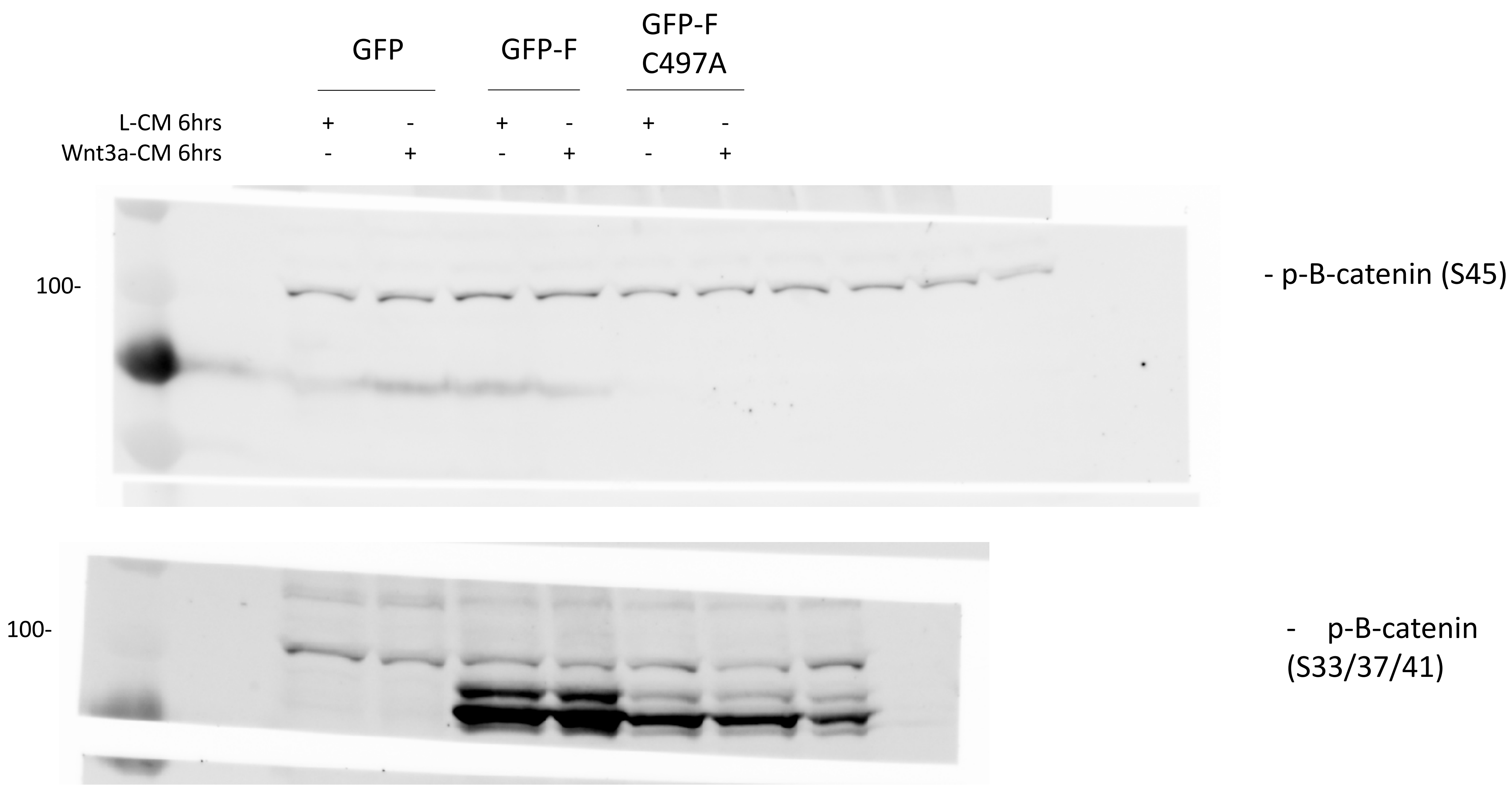

Supplementary Figure 11B.

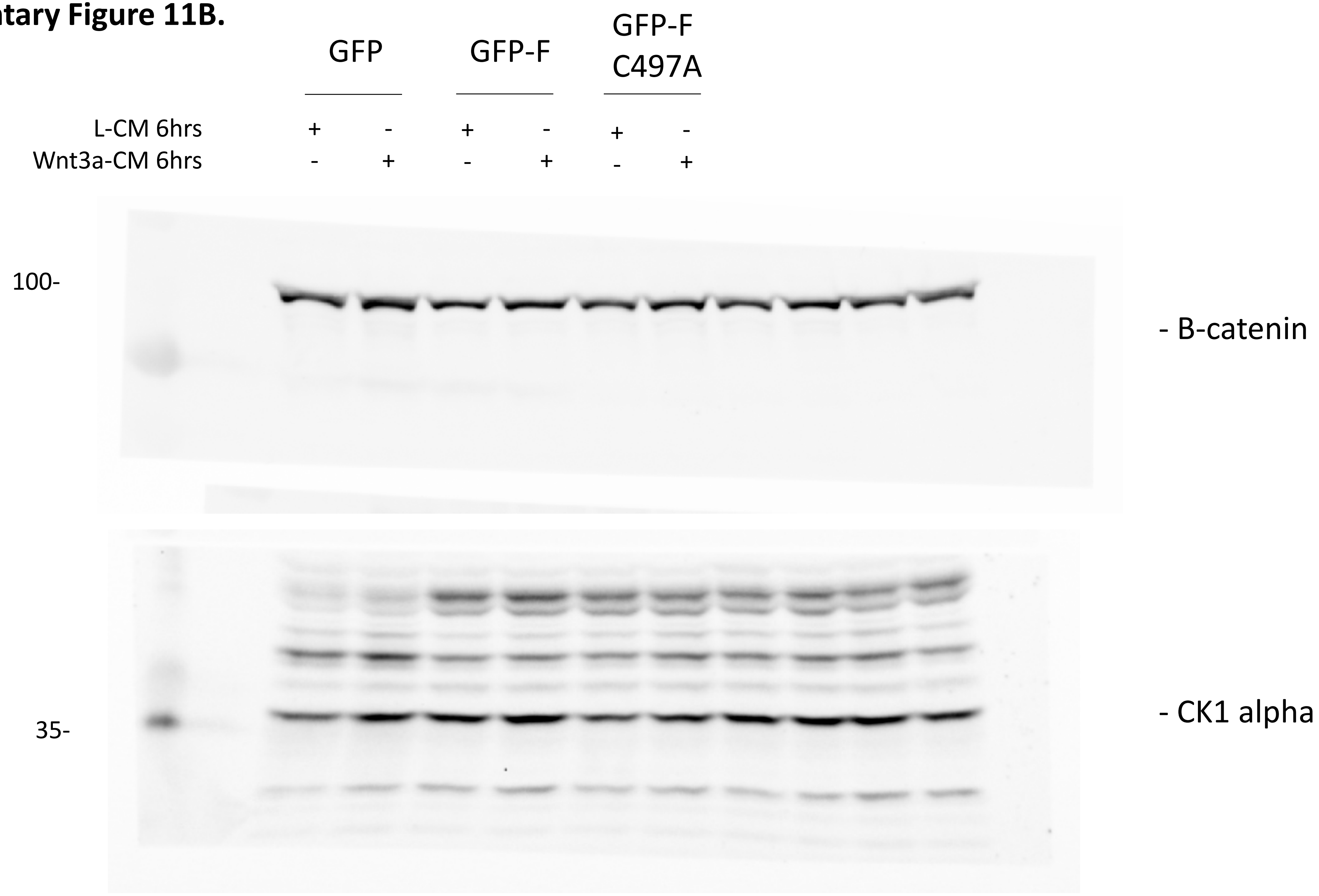

Supplementary Figure 11B.

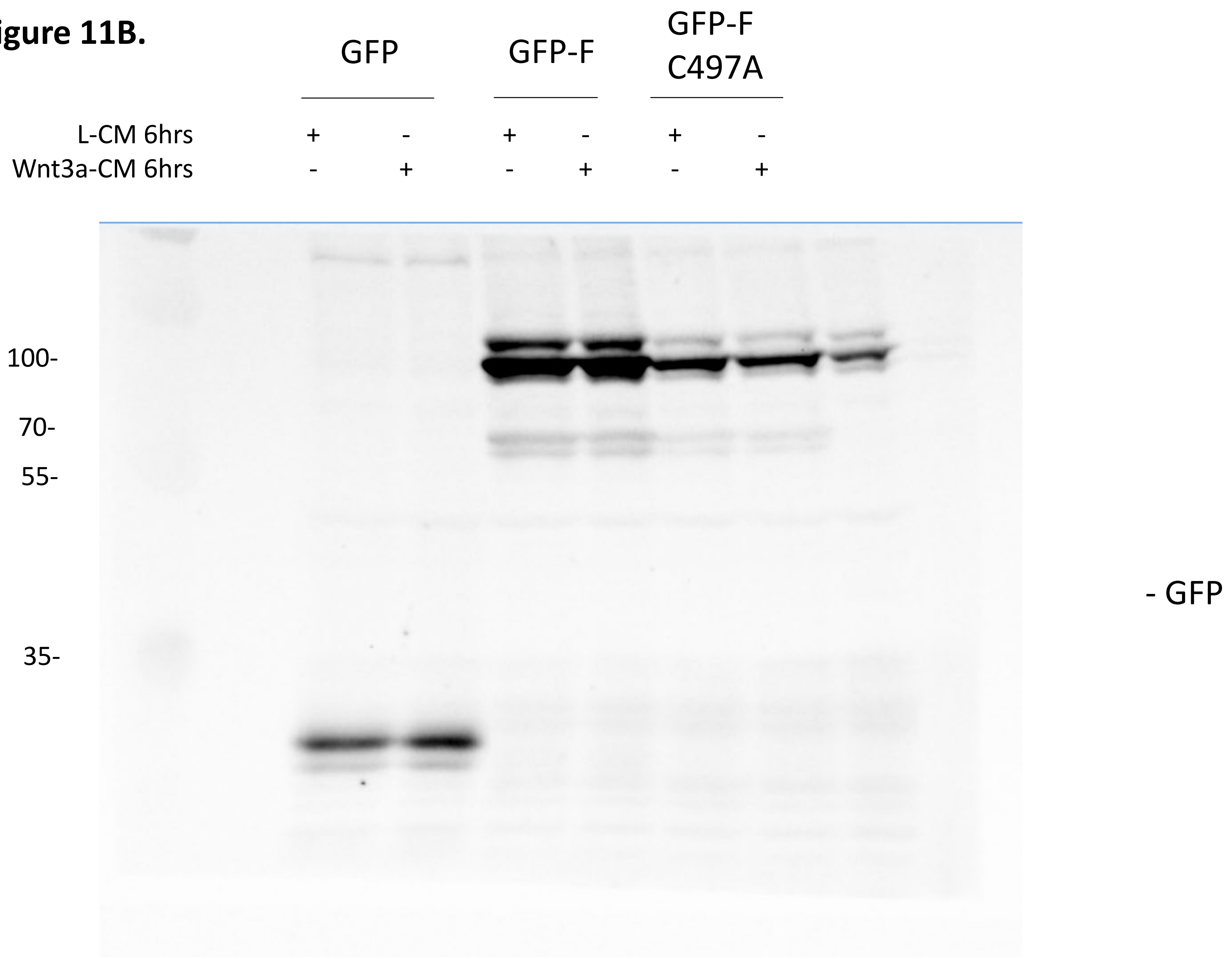

Supplementary Figure 11B.

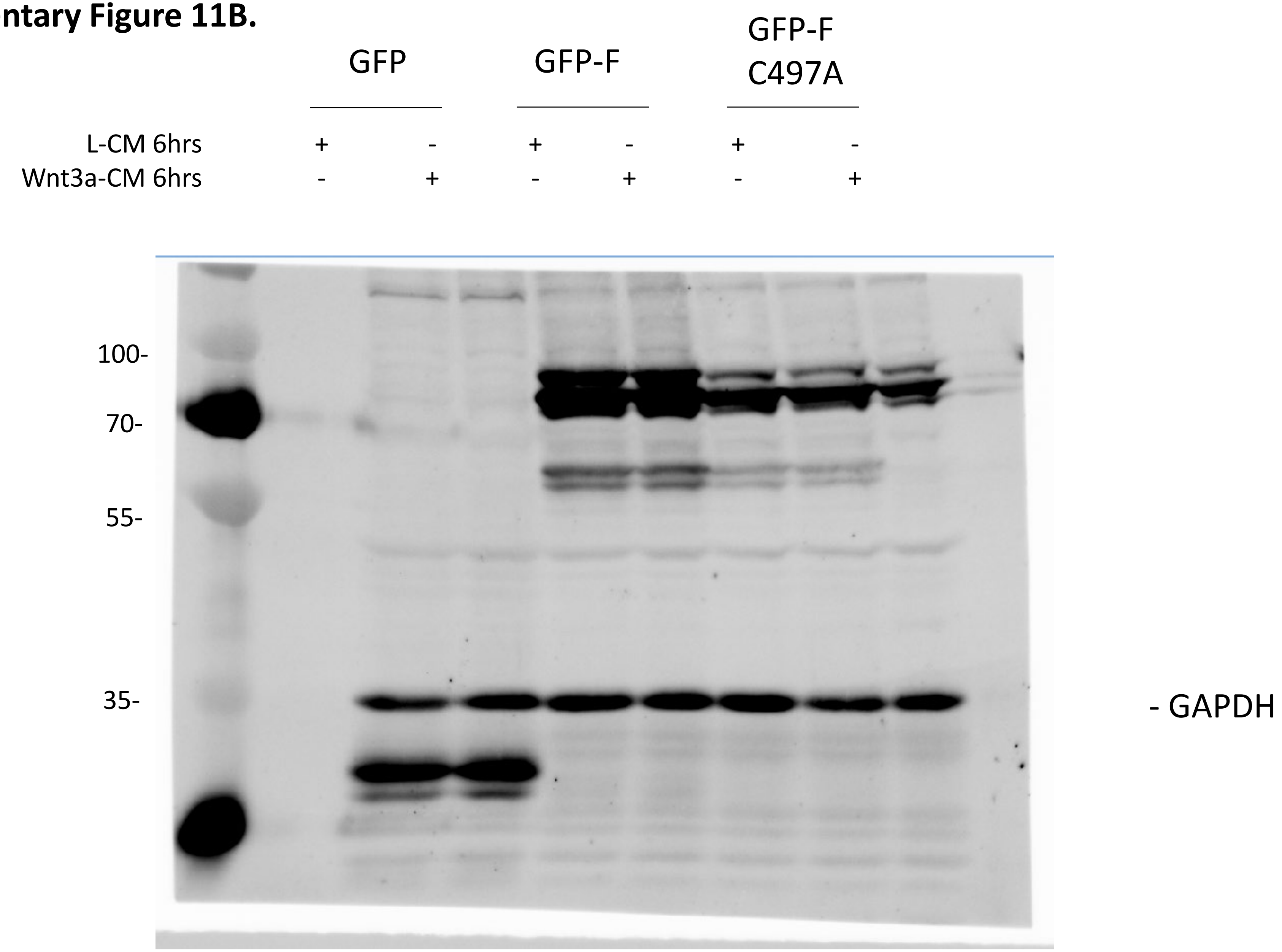

Supplement: Supplementary file 12 [file LSA-2020-00805_SdataFS11.pdf]
